# Enhanced Formation of Brominated and Nitrogenous Disinfection Byproducts in Drinking Water Disinfection with Chlorocyanurates

**DOI:** 10.1021/acs.est.5c07394

**Published:** 2025-12-19

**Authors:** Kadmiel B. Adusei, Hafiz Usama Tanveer, Zachary T. Kralles, Kirin Emlet Furst

**Affiliations:** † Charles E. Via, Jr. Department of Civil and Environmental Engineering, Occoquan Watershed Monitoring Laboratory, 1757 Virginia Polytechnic Institute and State University, Manassas, Virginia 20110, United States; ‡ Sid and Reva Dewberry Department of Civil, Environmental and Infrastructure Engineering, 3298 George Mason University, Fairfax, Virginia 22030, United States; § Department of Environmental Health and Engineering, 1466 Johns Hopkins University, Baltimore, Maryland 21205, United States

**Keywords:** Disinfection byproducts, drinking water treatment, alternative disinfectants, chlorine, cyanuric acid, toxicity

## Abstract

Disinfection of drinking water provides essential protection against microbial pathogens. However, disinfectants react with organic matter and other constituents in water to form disinfection byproducts (DBPs), which are of concern for human health. Chlorocyanurates are chlorine-based disinfectants that have been used for drinking water in point-of-use and emergency contexts. Little is known about chlorocyanurate DBP formation beyond the potential to form lower regulated trihalomethanes and haloacetic acids compared to chlorine. In this study, regulated and unregulated DBP formation was evaluated for multiple chlorocyanurate formulations to understand the effect of the chlorine-to-cyanuric acid ratio on DBP mixture composition and calculated toxicity by comparison to conventional chlorine. Chlorocyanurates produced lower regulated DBPs by ∼10–50% compared to chlorine but promoted bromine incorporation in most DBP classes by 50–200% and produced higher calculated toxicity than chlorine under most conditions. Enhanced dichloroacetonitrile formation by chlorocyanurates was partly attributed to trichloramine formation from the degradation of chlorocyanurates by hypochlorite. Thus, chlorocyanurates may promote multiple DBP toxicity drivers. Water quality and operational considerations are identified to minimize DBP toxicity while using chlorocyanurate disinfectants, which remain an important option for drinking water disinfection in low-resource settings.

## Graphical abstract

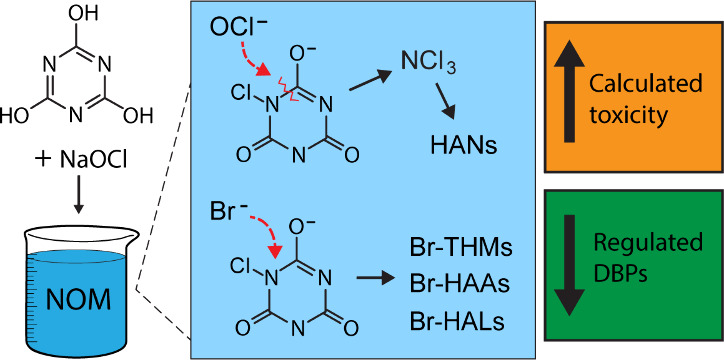

## Introduction

Disinfection of drinking water is practiced to protect against microbial pathogens and has significantly reduced mortality from waterborne diseases.^1,2^ However, disinfectants react with dissolved organic matter and other constituents in water to form disinfectant byproducts (DBPs).^3^ Over 700 DBPs have been detected in drinking water,^4^ some of which are associated with increased risk of cancer^5^ and adverse reproductive outcomes.^6^ International guidelines and national regulations focus on limiting the four bromo- and chloro-trihalomethanes (THMs) and a subset of haloacetic acids (HAAs) as indicators of the overall DBP mixture.^7,8^ Certain unregulated DBPs, e.g., haloacetonitriles (HANs), have particularly high toxic potency and may drive DBP mixture toxicity in some waters.^9^

Chlorine, the most common disinfectant, is effective, easy to use, and leaves a residual that protects against pathogen reintroduction.^10,11^ However, chlorine is associated with high regulated DBP levels compared to common alternatives.^12,13^ Furthermore, water systems with large service areas, high chlorine demand, intermittent operation, or high temperatures can have difficulty maintaining chlorine residuals.^14,15^ Monochloramine is the most popular alternative to chlorine in the U.S., as it tends to form lower levels of regulated DBPs and provides a more stable residual.^16^ However, implementing monochloramine is challenging for most small, rural, or low-income water systems due to dosing complexity, nitrification risk, and additional monitoring requirements.^17^ Thus, there is a need for alternative disinfectants that are easy to manage, provide a more stable residual than chlorine, and minimize DBP formation without compromising protection against pathogens.

Chlorocyanurates (also known as chloroisocyanurates or chlorinated cyanurates) are a class of chlorine-based disinfectants that could address this need. Chlorocyanurates are formed by addition of cyanuric acid to chlorine, most commonly in a 1:2 or 1:3 molar ratio as solid formulations of sodium dichloroisocyanurate (dichlor) or trichloroisocyanuric acid (trichlor), respectively.^18^ Solid chlorocyanurate products feature longer shelf-lives than liquid chlorine (NaOCl) without the disadvantages of calcium hypochlorite.^19^ Dichlor has been widely used for point-of-use disinfection by households and in emergency settings.^20,21^ Several full-scale, rural U.S. water systems implemented dichlor, prompting clarification from the U.S. Environmental Protection Agency (EPA) that chlorocyanurates cannot be used for compliance with disinfection regulations because they interfere with free chlorine measurement by EPA-approved analytical methods.^22^ Despite the analytical hurdle, this indicates the potential for the full-scale implementation of chlorocyanurate disinfection, particularly in small, rural systems.

In an aqueous mixture of cyanuric acid and chlorine, cyanuric acid acts as reservoir of free chlorine (HOCl, OCl^–^ and Cl_2_), where HOCl is considered the active disinfectant.^23,24^ A portion of the chlorine is bound to one or more nitrogen atoms in the cyanuric acid ring. The distribution of chlorocyanurate and nonchlorinated cyanurate species present depends on the ratio of chlorine to cyanuric acid, as well as pH and temperature.^25^ As free chlorine is consumed and the chemical equilibrium disturbed, the chlorine-nitrogen bond is rapidly hydrolyzed to release more free chlorine.^18^ At any given time, the free chlorine concentration in a chlorocyanurate solution is lower than in a chlorine solution of the equivalent total chlorine concentration, producing speculation that chlorocyanurate disinfection could offer a more stable residual with lower DBP formation than conventional chlorine.^26,27^ If true, these would be significant advantages—as long as an adequate free chlorine residual is maintained for effective disinfection.^28,29^

Several studies compared DBP formation from disinfection with chlorocyanurates and chlorine with mixed results. Compared to chlorine alone, Feldstein et al. observed a 29% decrease in total THM levels with cyanuric acid;^30^ however, brominated THM levels increased, which is a concern due to their higher toxic potency.^31–33^ However, the molar ratios of cyanuric acid to chlorine used in this study (e.g., 7.5:1, 15:1) were substantially higher than those in drinking water applications. Lower THM levels were also observed in swimming pools disinfected with trichlor vs chlorine.^34^ A study comparing THM formation from dichlor tablets and conventional chlorine in six drinking water sources in rural Tanzania found inconsistent differences.^35^ Dichlor formed THM levels that were lower (∼20%) or equivalent to chlorine in five waters but ∼30% higher in the sixth water, with no explanation. A more controlled study with one surface water found little difference in THM levels with dichlor and trichlor compared to chlorine, but 24% and 18% lower HAA5 levels with dichlor and trichlor, respectively.^25^

Despite decades of use of chlorocyanurates in drinking water, the formation of unregulated DBPs from these disinfectants has not been evaluated. Differences in formation pathways may produce divergent trends in unregulated DBP formation compared to those of THMs or HAAs during chlorocyanurate disinfection. There are several reasons to suspect that chlorocyanurates could increase the level of formation of unregulated DBPs of toxicological concern relative to conventional chlorine. First, if cyanuric acid addition increases the bromine substitution factor (BSF) of THMs in bromide-containing waters, BSFs of other DBP classes may also increase as well. While brominated THMs are associated with higher bladder cancer rates than chlorinated species,^36^ unregulated brominated HAAs and haloacetaldehydes (HALs) have even higher *in vitro* toxic potency.^9,37^ Second, Wahman et al. concluded that chlorocyanurates may engage in reactions beyond chlorine hydrolysis.^25^ Chlorocyanurate reaction pathways could promote the formation of specific DBP classes. For example, under certain conditions, cyanuric acid may decompose to form trichloramine,^38,39^ which can react with organic precursors to form nitrogenous DBPs like HANs.^40^ HANs have particularly high toxic potency, and may be key toxicity drivers in some source waters, particularly those impacted by wastewater.^13,14,41–43^ Understanding whether and how chlorocyanurates promote these DBPs of concern is necessary to balance public health trade-offs in drinking water disinfection.

This study evaluates the effect of chlorocyanurate disinfection on DBP formation and potential mixture toxicity and identifies mechanisms specific to this class of disinfectants. Twenty-four regulated and unregulated DBPs from six classes were measured in simulated distribution system (SDS) experiments with real and synthetic source waters disinfected with chlorine-only or chlorine with cyanuric acid in 3:1, 2:1, and 1:1 Cl:Cy molar ratios. These Cl:Cy molar ratios span a feasible range for drinking water applications, including the two commercially available formulations, and enable evaluation of DBP formation as a function of the cyanuric acid concentration. Water quality conditions that exacerbate or minimize DBP mixture toxicity with addition of cyanuric acid were identified. To the best of our knowledge, this study is the first to measure unregulated DBPs and evaluate DBP-associated toxicity in chlorocyanurate disinfection. By interrogating the effect of Cl:Cy ratio and interactions with pH and bromide, the findings produce novel insights into chlorocyanurate chemistry and indicate practical considerations for avoiding excess DBP risk in point-of-use and full-scale drinking water treatment.

## Materials and Methods

### Reagents and Chemicals

Cyanuric acid (98% purity) was purchased from Sigma-Aldrich. Reagent-grade sodium hypochlorite (NaOCl) was purchased from Fisher Scientific and standardized every 3 months by direct chlorine analysis at 292 nm with an Agilent Cary 60 UV–vis spectrophotometer. All other reagents, solvents, and reference standards were purchased of the highest available purity and are described in Text S1 and Table S2. Glassware and utensils were precleaned following the chlorine-demand free protocol. NaOCl and cyanuric acid working stock solutions were prepared fresh in DI water (Milli-Q Direct 8) each day. Chlorocyanurate working stocks were prepared from chlorine and cyanuric acid solutions following previously reported methods^44,45^ to ensure consistent dosing for chlorine and chlorocyanurate conditions and enable testing of Cl:Cy molar ratios beyond available commercial products.

### Sample Preparation and Water Quality Analysis

Surface water samples were collected downstream of a reservoir for an indirect potable reuse system. Samples were collected in summer 2023, winter 2023, and fall 2024 to capture seasonal variation in the precursor content. Sample pH and conductivity were measured with a Hanna multimeter. Samples were collected in chlorine-demand free, 1-L PTFE bottles, vacuum-filtered with 0.7-μm glass fiber (GF/F) filters (Whatman), and stored at 4 °C until analysis. Dissolved organic carbon (DOC) was analyzed in filtered samples with a Shimadzu TOC-L Analyzer. Absorbance at UV_254_ was measured with an Agilent Cary 60 UV–vis spectrophotometer and divided by the DOC concentration to calculate SUVA_254_, an indicator of DOC aromaticity.^46^ Bromide was measured with a Dionex Integrion Ion Chromatograph. Each water sample was split into a “low bromide” condition (no additional bromide added) and a “high bromide” condition amended with 100 μg/L bromide prior to disinfection.

### Simulated Distribution System Disinfection Experiments

Simulated Distribution System (SDS) experiments were conducted following Furst et al.^7,47^ with real and synthetic source waters. Synthetic samples were prepared with DI water buffered to pH 7.3 with 10 mM potassium dihydrogen phosphate and disodium hydrogen phosphate and amended with 5-mg/L humic acid (Sigma-Aldrich), manufactured in Switzerland, as a model DBP precursor. In synthetic samples and other buffered experiments, pH was monitored to ensure a lack of drift. Chlorine (NaOCl) working solution (16.9 mM) was prepared in DI water alone or with cyanuric acid targeting Cl:Cy molar ratios of 1:1 (monochlor), 2:1 (dichlor), and 3:1 (trichlor). Cyanuric acid was dissolved in DI water and stirred for 20 min prior to addition of NaOCl. The 24-h chlorine demand was determined for each sample.^48^

SDS experiments were conducted in 60 mL headspace-free vials, spiked in triplicate to target 24 h total chlorine residuals of 1 mg/L as Cl_2_, and held in the dark at room temperature. After 24 h, pH was measured and total chlorine analyzed using N,N-diethyl-p-phenylenediamine (DPD). Average free, total, and combined chlorine concentrations for all surface water and synthetic water experiments are provided in Table S11. Total chlorine residuals were within 0.56 and 1.4 mg/L as Cl_2_. Combined chlorine concentrations were below 0.2 mg/L in all experiments, as the DPD method cannot distinguish between chlorine and chlorocyanurates.^45^ No significant differences in residuals were identified between disinfectant conditions within each experiment (Wilcoxon rank sum test, *p* > 0.05). Residuals were quenched with 33 mg/L ascorbic acid and samples stored at 4 °C until analysis within 24 (volatiles) or 48 h (HAAs). The concentrations of chlorine and cyanurate species were calculated using reported equilibrium constants.^49,50^ At environmental pH, aqueous cyanuric acid (H_3_Cy) undergoes stepwise deprotonation to H_2_Cy^–^ and HCy^2–^, and up to six species of chlorocyanurates form by rapid reactions with free chlorine: Cl_3_Cy, HCl_2_Cy, H_2_ClCy, HClCy^–^, C1_2_Cy^–^, and ClCy^2–^ (Figure S1).

### DBP Analysis

Fifteen volatile and semivolatile DBPs were measured, including THMs: chloroform (TCM), bromodichloromethane (BDCM), dibromochloromethane (DBCM), and bromoform (TBM); HANs: trichloroacetonitrile (TCAN), dichloroacetonitrile (DCAN), bromochloroacetonitrile (BCAN), and dibromoacetonitrile (DBAN); HALs: trichloroacetaldehyde (TCAL), bromodichloroacetaldehyde (BDCAL), dibromochloroacetaldehyde (DBCAL), and tribromoacetaldehyde (TBAL); haloketones (HKs): 1,1,1-trichloropropanone and 1,1-dichloropropanone; and trichloronitromethane (TCNM). Nine HAAs were measured: chloroacetic acid (CAA), bromoacetic acid (BAA), dichloroacetic acid (DCAA), bromochloroacetic acid (BCAA), dibromoacetic acid (DBAA), trichloroacetic acid (TCAA), bromodichloroacetic acid (BDCAA), dibromochloroacetic acid (DBCAA), and tribromoacetic acid (TBAA). Volatile DBPs were extracted using modified EPA Method 551.1 as described by Zeng et al.^51^ Briefly, triplicate 50 mL samples were dosed with 3 mL of methyl-*tert*-butyl ether (MtBE) containing internal standard (IS) 1,2-dibromopropanone (1,2-DBP) for quantitation. HAAs were extracted with methyl ester-derivatization.^51^ Triplicate 50 mL samples were acidified to pH < 1 with 1 mL 98% sulfuric acid prior to addition of 4 mL MtBE containing the IS and surrogate (2-bromobutyric acid) for evaluating recovery, followed by 12-g dehydrated sodium sulfate (NaSO4). Following 2 min of shaking, the MtBE extracts were transferred to vials containing ∼0.5 g NaSO_4_ for dehydration before evaporating to 1 mL under a gentle stream of nitrogen. Extracts were stored at −20 °C and analyzed within 10 days.

Analysis was performed with an Agilent 7010*b*/8890 triple quadrupole gas chromatography mass spectrometer (GC-MS/MS) with a high efficiency electron ionization (EI) source operated in Multiple Reaction Monitoring (MRM) mode. Separation was performed with an Rtx-200MS GC column (30 m × 0.25 mm × 0.25 μm) from Restek Corporation. Instrumental parameters are provided in Text S2. Optimized MRM transitions, collision energies, and GC retention times are listed in Table S3. Method reporting limits (MRLs) ranged from 0.003 to 0.017 μg/L, and analyte recoveries were within ∼70 to 130% (Table S4).

### Calculated Toxicity

Toxicity-weighted concentrations were calculated from measured DBP concentrations to compare relative toxicity of DBP mixtures formed by each disinfectant condition, following methods used previously for comparing relative toxicity of DBPs formed by chlorine and chloramine.^41,52,53^ Briefly, the measured concentration of each DBP (excluding haloketones) was divided by the LC_50_ chronic cytotoxicity index values derived from *in vitro* assays with Chinese Hamster Ovary (CHO) cells, as published by Wagner and Plewa.^9^ These toxic-potency-weighted concentrations were summed to produce cumulative toxic-potency-weighted concentrations for each DBP class and for all measured DBPs. The assumption of additivity was supported by the findings of Lau et al.^54^ The CHO chronic cytotoxicity assay screens for a broad range of toxicity end points using a mammalian model but is not representative of human health risk. Until human health guideline values are available for more DBP species, this approach enables relative comparisons of the toxicity of the DBP mixtures formed under different treatment conditions.

### Density Functional Theory (DFT) Model

Chlorocyanurates were drawn using ACD/ChemSketch (version 2021.2.1) and exported as. mol files. These were imported into Avogadro (version 1.2.0) to generate input (.inp) files for the ORCA. Single-point energy calculations were performed using density functional theory (DFT) with the def2-SVP basis set (multiplicity = 1). ORCA (version 6.0.1) was used to compute electrostatic potential (ESP) values, which were saved as Gaussian-type .cube files. Quantitative descriptors, including minimum, maximum, and average ESP values, and ESP-mapped surface area, were calculated from the .cube files using Multiwfn.^55,56^

## Results and Discussion

### Effect of Cl:Cy ratio on DBP formation

Surface water samples were collected downstream of a reservoir that serves as the environmental buffer for an indirect potable reuse system. Sample collection was conducted in the summer and winter of 2023 and fall of 2024 to capture seasonal variation. Water quality varied moderately between seasons by pH (7.3–8.4), DOC (5.0–8.0 mg/L) and SUVA254 (1.3–4.6 L/mg-m) (Table S1). Conductivity varied from 0.27 and 0.42 μS/cm in the summer and fall samples, respectively, and increased to 1.1 μS/cm in the winter sample. Similarly, bromide was low in summer and fall (0.04–0.05 mg/L) but increased dramatically in the winter of 2023 to 0.96 mg/L; thus, the winter sample is evaluated as a high bromide condition. Chlorine demand (24-h) was consistent between seasons (∼12 mg/L as Cl_2_). Chlorine residuals and DBP concentrations for all surface water SDS experiments are reported in Table S6.

DBP concentrations were measured following disinfection by chlorine-only or chlorine with cyanuric acid in 3:1 (trichlor), 2:1 (dichlor), or 1:1 (monochlor) molar ratios. Trichlor is the highest Cl:Cy ratio used in practice, while dichlor is most used. Monochlor represents a lower Cl:Cy ratio that may be relevant for drinking water applications, although it is primarily included to understand how DBP formation is affected by chlorocyanurate speciation. The total DBP concentration in the fall sample treated by chlorine was 115 μg/L; this substantially declined with cyanuric acid addition by 27% (trichlor), 35% (dichlor) and 45% monochlor (Figure 1A). The THM concentration was highest with chlorination (46 μg/L) and decreased by 29% with a low dose of cyanuric acid (trichlor). With further cyanuric acid addition, THM concentrations declined by 31% (dichlor) and 49% (monochlor) compared to chlorine-only. The HAA concentration, 66 μg/L with chlorine, also declined with increasing cyanuric acid by 26% (trichlor), 40% (dichlor) and 44% (monochlor). As with THMs and HAAs, HALs and TCNM concentrations decreased with increasing cyanuric acid addition. By contrast, HAN concentrations substantially increased from chlorine (0.71 μg/L) by 88% with trichlor and remained elevated by 86% (dichlor) and 61% (monochlor). Only HKs also increased with cyanuric acid addition, from 0.22 μg/L with chlorine up to 216% with monochlor. However, HK concentrations remained <1 μg/L across samples and their toxicity is unknown; thus, they are not further discussed.

**Figure 1:**
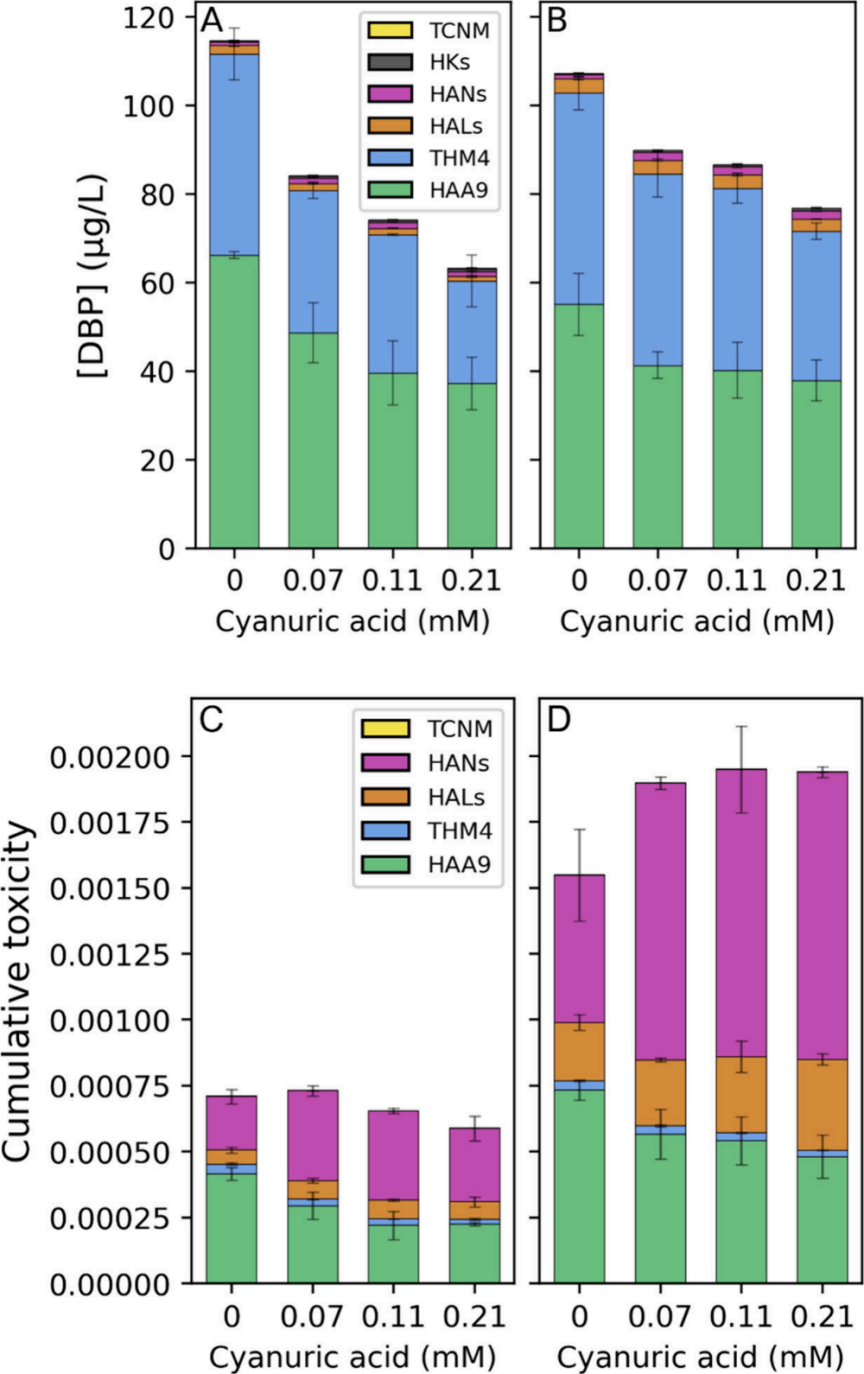
DBP concentrations in fall 2024 samples on a weight basis with (A) low (0.05 mg/L) bromide or (B) moderate (0.15 mg/L) bromide and toxic potency-weighted basis with (C) low or (D) moderate bromide, following treatment with 0.21 mM chlorine-only or with cyanuric acid.

In the summer sample, cyanuric acid addition had similar effects on DBP formation (Figure S2A). As HAAs were not measured in summer, only volatile DBPs are directly comparable with fall. With increasing cyanuric acid addition, total volatile DBPs declined by 5% (trichlor), 9% (dichlor), and 35% (monochlor) relative to chlorine. Summer volatile DBP concentrations were significantly higher than fall, consistent with higher SUVA_254_ (4.9 vs 1.3 L/mg-m).^57^ Comparing chlorinated samples, summer THM concentrations (94 μg/L) nearly doubled and HAL concentrations (11 μg/L) were more than five times higher than fall concentrations (Figure S2A). The summer HAN concentration (3.4 μg/L) was also higher than fall (0.7 μg/L). When treated with trichlor and dichlor, summer THM concentrations (86 and 81 μg/L) decreased by 9% and 14% compared to chlorine, smaller declines than observed in fall. With monochlor, however, summer THM concentrations decreased by 44%, similar to fall. Summer HAL concentrations also decreased with cyanuric acid addition, though less dramatically than in fall, by 12% (trichlor), 19% (dichlor), and 22% (monochlor). As in fall, summer HAN concentrations substantially increased with cyanuric acid addition by 58% (trichlor), 60% (dichlor) and 52% (monochlor) compared to chlorine-only. Thus, across both seasons, cyanuric acid addition resulted in a progressive decrease in most DBP classes by as much as ∼50%, while HANs increased by ∼50–90%. Lower THM levels with chlorocyanurate disinfection compared to conventional chlorine was previously reported, but the finding that cyanuric acid can promote certain DBP classes is new. Increased HAN concentrations are particularly concerning due to their relatively high toxic potency.

To evaluate the potential impact of chlorocyanurate disinfection on mixture toxicity, DBP concentrations were divided by their LC_50_ values. Although total DBP concentrations substantially declined with cyanuric acid addition in the fall, total calculated toxicity only modestly declined as increased HAN-associated toxicity compensated for lower HAA-associated toxicity (Figure 1C). The HAN calculated toxicity increased by 68% (trichlor), 67% (dichlor) and 38% (monochlor) compared to chlorine in fall, and to a lesser extent in summer (39–38%). In summer, the HAL calculated toxicity was higher than in fall and increased substantially with cyanuric acid addition, by 73% (trichlor), 90% (dichlor), and 154% (monochlor) compared to chlorine (Figure S2C). This is driven by the shift with higher cyanuric acid addition toward brominated HALs, which have higher toxic potency than TCAL. Interestingly, brominated HAN concentrations did not increase. The differentiating effects of bromide on chlorocyanurate DBP formation pathways are investigated further in the following section.

### Combined Effects of Bromide and Cl:Cy Ratio on DBP Formation

In the summer and fall experiments with low bromide levels (≤0.05 mg/L), cyanuric acid increased bromine substitution for several DBP classes. It would be concerning if this trend is amplified at higher bromide levels, as brominated DBP species generally exhibit higher cytotoxicity than their chlorinated counterparts.^58^ To investigate the effect of bromide on chlorocyanurate DBP formation and toxicity, the summer and fall samples were supplemented with 100 μg/L bromide prior to disinfection. The resulting total bromide concentrations (summer: 140 μg/L, fall: 147 μg/L) represent ∼90th percentile bromide concentrations among U.S. water systems.^8^ The winter 2023 sample (960 μg/L) was evaluated as a high bromide condition, representing the ∼99th percentile bromide level among U.S. water systems.^8^

With addition of moderate bromide in fall, total DBP concentrations slightly decreased with chlorine (6%) but moderately increased with cyanuric acid addition to 7% (trichlor), 17% (dichlor), and 21% (monochlor) (Figure 1). The increase was primarily driven by THMs, which increased by 34% (trichlor), 31% (dichlor) and 45% (monochlor) compared to low bromide. By contrast, HAA concentrations decreased with bromide addition, by 17% and 15% with chlorine and trichlor respectively, and exhibited no difference with dichlor or monochlor. In summer, the addition of moderate bromide also resulted in higher THM concentrations with chlorine (18%) and monochlor (30%), but effectively no change with trichlor or dichlor (Figure S2B). The combination of moderate bromide and monochlor produced the greatest increase in volatile DBP mass in both the fall (49%) and summer (30%). In the winter sample with high bromide, the effect of cyanuric acid addition on total DBP concentrations was similar in magnitude to summer or fall (Figure S3).

With moderate bromide addition in the fall and summer, HAL concentrations increased across all disinfection conditions compared to the low bromide scenarios. However, bromide increased HAL concentrations significantly more under chlorocyanurate conditions, suggesting that bromide directly interacts with cyanurate species. This was especially true in fall, where moderate bromide addition increased HAL concentrations by 70% (chlorine), 103% (trichlor), 117% (dichlor) and 159% (monochlor) compared to low bromide (Figure 1B). In the summer, moderate bromide addition did not change HAL concentrations with chlorine and resulted in more modest increases with trichlor (11%), dichlor (13%), and monochlor (16%) (Figure S2B). In the high bromide winter sample, HAL concentrations in all chlorocyanurate conditions surpassed chlorine. Trichlor had the highest HAL concentration, 177% greater than that of chlorine (Figure S3A). In all seasons, but particularly fall and winter, brominated HALs drove the increase in total HAL concentrations with cyanuric acid addition.

HAN formation was also promoted by moderate bromide in both the fall and summer; in most cases, this effect was enhanced by cyanuric acid addition. In fall, the addition of moderate bromide resulted in increased HAN concentrations by 23% with chlorine, and by 36%, 42%, and 68% with trichlor, dichlor and monochlor, respectively (Figure 1B). In summer, moderate bromide addition also increased HAN levels—though as with HALs, the difference between chlorine (22%) and chlorocyanurates (29–23%) was less distinct (Figure S2B). Without bromide addition, the summer sample had elevated HAN and HAL levels compared with fall, suggesting temporal variation in the chlorine reactivity of the precursor pool. A more extensive sampling campaign would be needed to determine whether this variation represents a seasonal pattern. In the high bromide winter sample, HAN concentrations were also promoted with cyanuric acid addition by 66% (trichlor), 29% (dichlor), and 24% (monochlor).

Across all bromide levels and seasons, chlorocyanurates significantly promoted BSFs of most DBP classes relative to chlorine (Figure 2). For THMs, di-HAAs and tri-HAAs, BSFs progressively increased with cyanuric acid addition by up to ∼50% with monochlor compared to chlorine. However, cyanuric acid promoted HAL BSFs to a much greater extent. In fall with low bromide, HAL BSFs increased by 98% (trichlor), 110% (dichlor), and 173% (monochlor) compared to chlorine. With moderate bromide, HAL BSFs increased by 53% (trichlor), 46% (dichlor), and 105% (monochlor) compared to chlorine. Even greater increases in HAL BSFs were observed with cyanuric acid addition in summer, by 198% (low bromide) and 157% (high bromide) with monochlor compared to chlorine. In the high bromide winter sample, HAL BSFs also increased substantially with cyanuric acid addition by 50–65% compared to chlorine.

**Figure 2:**
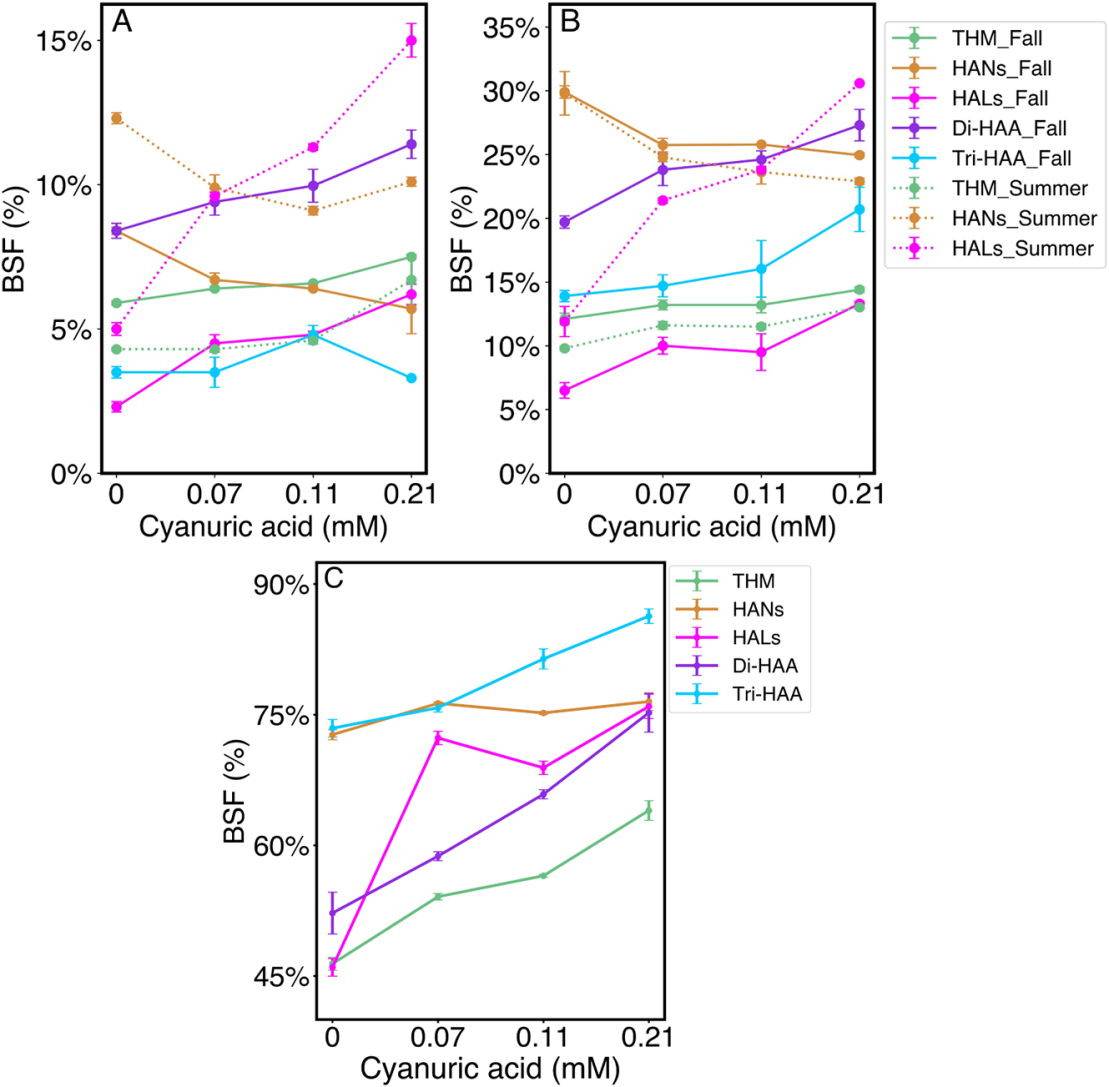
Bromide substitution factors (BSFs) expressed as a percent for DBP classes measured in fall and summer samples with (A) low bromide (0.05 mg/L) or (B) moderate bromide (0.15 mg/L) and (C) high bromide (0.96 mg/L) winter sample, in SDS experiments with 0.21 mM chlorine-only or with cyanuric acid in 3:1, 2:1, and 1:1 Cl:Cy molar ratios.

Remarkably, unlike all other DBP classes, HAN BSFs decreased or remained constant with the addition of cyanuric acid addition. This was true across all seasons and bromide conditions (Figure 2). Under low bromide conditions in fall and summer, HAN BSFs under chlorocyanurate conditions were ∼20–30% less than with chlorine. In fall and summer with moderate bromide, HAN BSFs in chlorocyanurate conditions were ∼15–25% less than with chlorine. In the high bromide winter sample, HAN BSFs remained essentially constant (≤5%) with cyanuric acid addition. These findings are particularly interesting because HAN formation was promoted by increasing bromide and cyanuric acid addition but not HAN BSFs. This suggests that HANs are formed and brominated via distinct pathways in the presence of cyanurates compared to other DBP classes.

In conventional chlorine systems, bromide reacts with free chlorine (HOCl) to form bromine (HOBr).^59,60^ Chlorocyanurate systems feature lower free chlorine levels, particularly at low Cl:Cy ratios (e.g., monochlor) in which <10% of the total chlorine is present as free chlorine (Table S5). Lower free chlorine results in higher effective bromide-to-chlorine ratios such that a higher percentage of free chlorine is converted to bromine. However, as free chlorine is consumed, cyanurate-bound chlorine is rapidly released.^61^ The HOBr concentration would be unaffected by the Cl:Cy ratio if the rate of chlorine hydrolysis is comparable to the reaction rate between HOCl and bromide (1550 M^–1^ s^–1^).^62^ Yet another bromination pathway may occur in chlorocyanurate systems. Chlorocyanurates are readily brominated through halogen substitution reactions with oxidized bromine species.^63,64^ Notably, tribromoisocyanurate has been used as a brominating agent for aromatic compounds.^65^ We hypothesize that bromocyanurates can act as brominating agents for aromatic DBP precursors, explaining why THM, HAA and HAL BSFs increase with cyanuric acid addition.

Conversely, HAN BSFs do not increase with cyanuric acid addition, suggesting that bromocyanurates are nonreactive with HAN precursors. Rather, we propose that HOBr is the brominating agent for HAN precursors in chlorocyanurate systems, as is thought to be the case with conventional chlorine. In high-bromide water, HAN BSFs were essentially constant across all chlorine and chlorocyanurate conditions, consistent with bromination of HAN precursors by an excess supply of HOBr. In the low and moderate bromide waters, however, HAN BSFs declined by ∼15–30% as a function of cyanuric acid addition; this is consistent with competition between chlorocyanurates and HAN precursors for limited HOBr.

For each season and disinfectant condition, bromide addition increased the cumulative calculated toxicity by promoting brominated DBP formation. Cyanuric acid addition further enhanced the calculated toxicity, particularly by promoting brominated HAL levels. In the fall sample with moderate bromide, the cumulative calculated toxicity for all chlorocyanurate conditions exceeded that of chlorine by ∼25% (Figure 1D). In the high bromide winter sample, the cumulative calculated toxicity in each chlorocyanurate condition was also higher than chlorine, by 74% (trichlor), 27% (dichlor), and 25% (monochlor) (Figure S3B). Thus, in the presence of moderate or high bromide, all chlorocyanurate conditions resulted in higher calculated toxicity than chlorine. HANs comprised most of the calculated toxicity in most waters, and contributed to the increase in calculated toxicity observed in chlorocyanurates vs chlorine, despite lower BSFs. As HANs and other nitrogenous DBPs may serve as toxicity drivers in DBP mixtures, it is important to understand the mechanisms by which chlorocyanurates promote HAN formation.

### Investigation of HAN Formation Mechanisms in Chlorocyanurate-Disinfected Waters

The experiments with real source waters demonstrate that chlorocyanurates can significantly promote HAN formation relative to conventional chlorine. To investigate the mechanisms by which this occurs, controlled experiments were conducted with synthetic waters. First, the reagents were ruled out as HAN sources by analyzing DI water amended with cyanuric acid only or with chlorine at each Cl:Cy ratio; no HANs were formed nor any other DBPs. Second, SDS experiments were conducted with synthetic source water consisting of 5 mg/L humic acid and 10 mM phosphate buffer at pH 7.3 to verify that HAN promotion by chlorocyanurates did not occur due to some unidentified constituents in the surface waters. DBP formation in synthetic water broadly exhibited the same trends observed in the real waters (Figure 3, Table S7). Though total DBP concentrations declined, HAN concentrations increased significantly with increasing cyanuric acid concentrations by 173% (trichlor), 200% (dichlor), and 45% (monochlor) compared to chlorine. Thus, chlorocyanurates promote HAN formation in the presence of chlorine and humic acid alone. The effect is most pronounced at higher Cl:Cy ratios (2:1 and 3:1).

**Figure 3:**
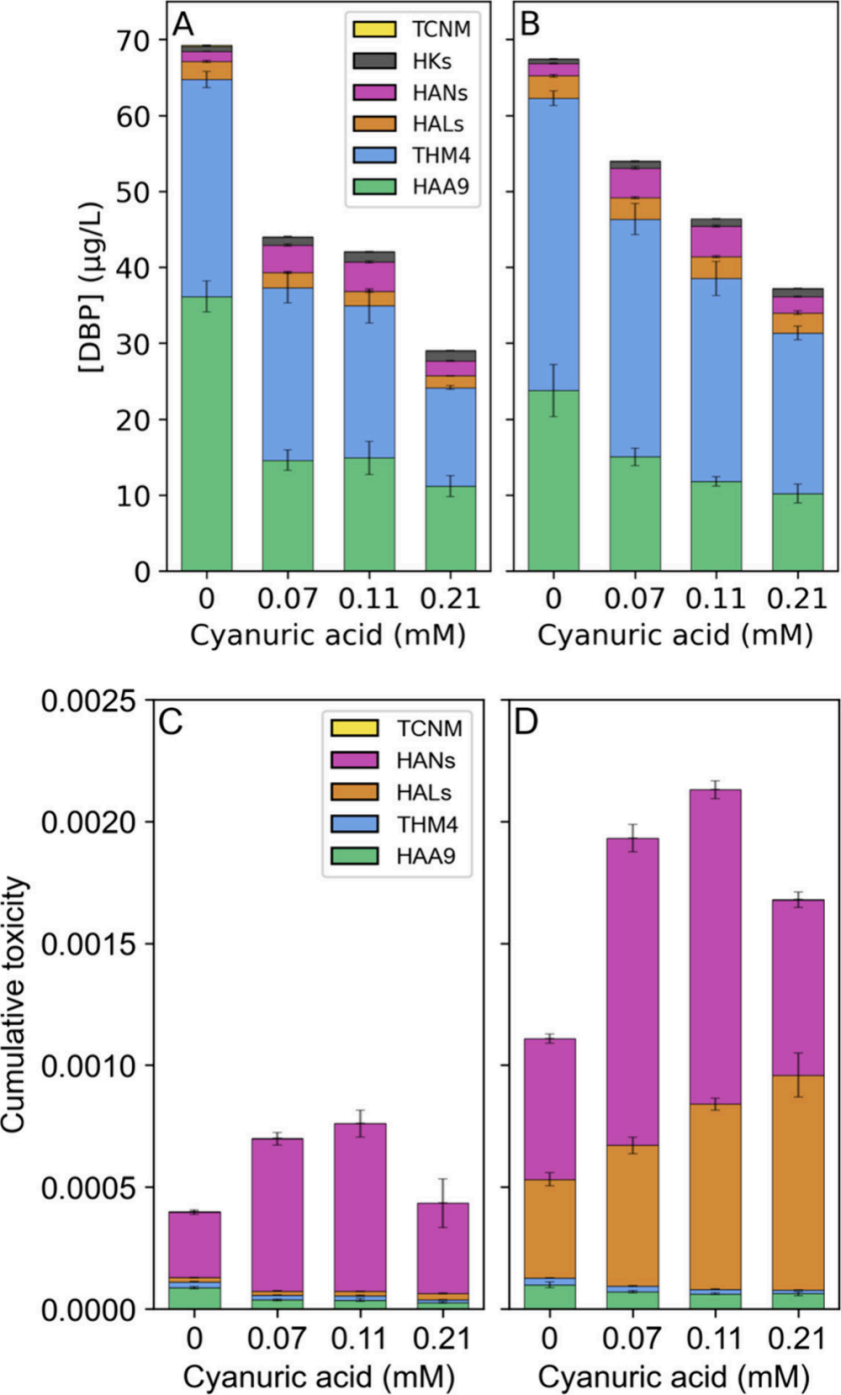
DBPs measured in synthetic water samples (pH 7.3, 5 mg/L humic acid), disinfected by 0.21 mM chlorine-only or with cyanuric acid in 3:1, 2:1, and 1:1 Cl:Cy molar ratios. DBP concentrations with (A) no bromide or (B) 100 μg/L bromide, and toxicity-weighted concentrations with (C) no bromide or (D) 100 μg/L bromide.

HAN accumulation in chlorocyanurate systems could be expected, with lower free chlorine levels producing slower HAN hydrolysis rates.^66,67^ However, if this were the only mechanism at play, HAN concentrations would be the highest with monochlor, which has the lowest free chlorine levels. A different mechanism must explain why trichlor and dichlor produce the highest HAN concentrations.

One potential explanation for the increased HAN formation is that chlorocyanurates can decompose to trichloramine,^38,39^ which subsequently reacts with organic matter to form HANs.^40^ Trichloramine formation in concentrated chlorocyanurate slurries was documented to occur in a patented dichlor production process.^68^ To determine whether trichloramine can form in more dilute chlorocyanurate solutions, experiments were performed with 18 mM NaOCl and cyanuric acid in 3:1, 2:1 or 1:1 Cl:Cy molar ratios at pH 7.3 or 9.2. Trichloramine was analyzed by absorbance at 336 nm (*ε*_336_ = 195 M^–1^ cm^–1^) and 360 nm (*ε*_360_ = 130 M^–1^ cm^–1^) with control for hypochlorite interference following Chuang et al. (Text S3).^69^

At pH 7.3, no trichloramine was observed. At pH 9.2 however, significant trichloramine formation was observed with trichlor and dichlor and a comparatively small amount with monochlor (Figure 4A). Trichloramine concentrations peaked within the first hour with dichlor and trichlor, reaching maxima of 277 μM and 230 μM respectively, and continued to slightly rise with monochlor, reaching 109 μM after 2.5 h. Cyanuric acid decay was confirmed using the melamine-induced turbidity method (Text S4). Molar yields of trichloramine from cyanuric acid estimated from the maximum concentrations are 3.4% with trichlor, 2.1% with dichlor, and 0.55% with monochlor (Table S10). These results provide a first indication of potentially significant trichloramine formation from chlorocyanurate decomposition at Cl:Cy ratios relevant for drinking water.

**Figure 4:**
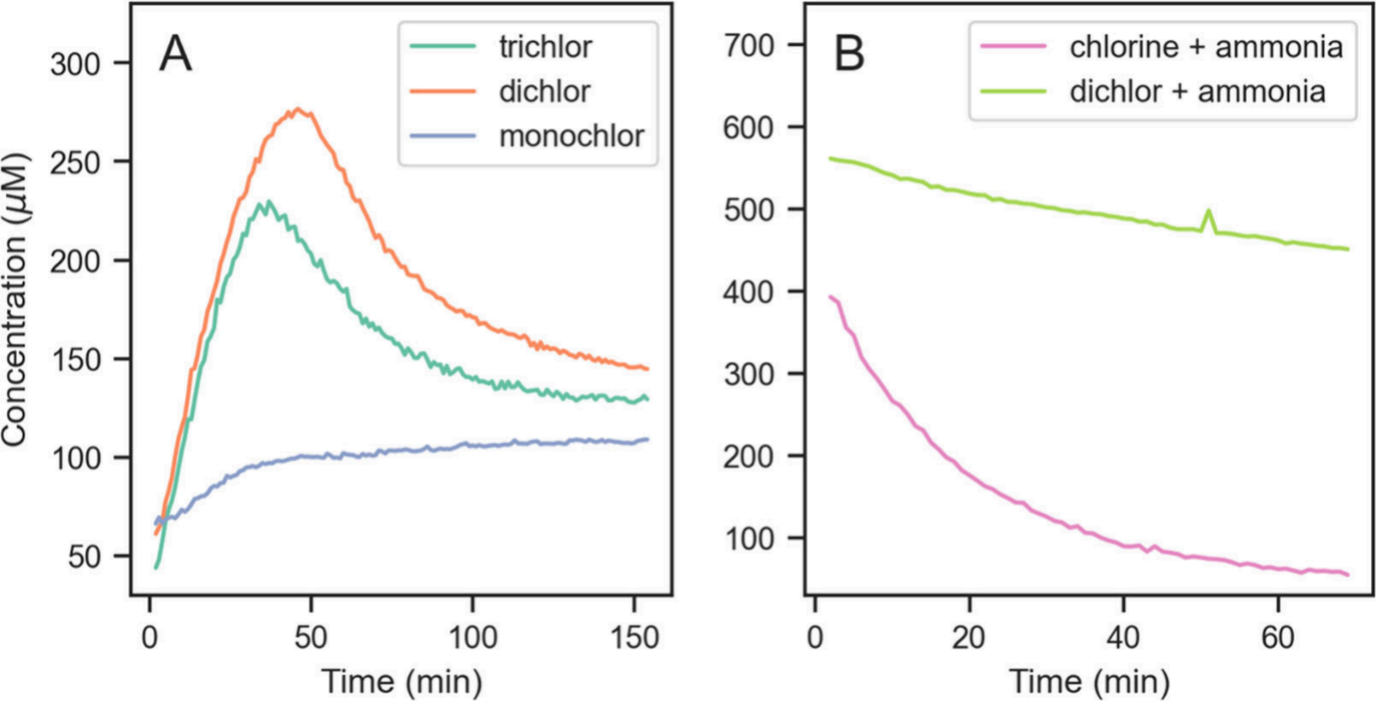
Concentrations of trichloramine (μM) formed in experiments with DI water and 18 mM chlorine at (A) pH 9.2 with cyanuric acid in Cl:Cy molar ratios of 3:1 (trichlor), 2:1 (dichlor), or 1:1 (monochlor), and (B) pH 7.2 with 1.4 mM ammonia and no cyanuric acid (“chlorine + ammonia”) or with cyanuric acid in Cl:Cy ratio of 2:1 (“dichlor + ammonia”).

These results are consistent with a patent documenting rapid cyanuric acid degradation by chlorine at high Cl:Cy ratios and high pH (e.g., 9–10).^68^ The effect of pH is also consistent with the pathway proposed by Wojtowicz^39^ for hydrolysis of dichloroisocyanurate by hypochlorite to form trichloramine (eq 1):

1 $${\mathrm{Cl}}_{2}{\mathrm{Cy}}^{-}+7{\mathrm{ClO}}^{-}+4H_{2}O\to 3{\mathrm{NCl}}_{3}+2{\mathrm{CO}}_{2}+{{\mathrm{HCO}}_{3}}^{-}+7{\mathrm{OH}}^{-}$$

The portion of free chlorine present as hypochlorite is ∼4-fold higher at pH 9 than at pH 7. Along with pH, the Cl:Cy ratio controls the distribution of chlorine and cyanurate species (Table S5). Trichlor has the highest equilibrium free chlorine concentration and therefore the highest hypochlorite concentration at any pH, while lower Cl:Cy ratios produce much lower hypochlorite concentrations. For example, at pH 9.2, the percent total chlorine present as hypochlorite in the trichlor condition (69.3%) is twice that of monochlor (34.5%) (Table S5).

The rate of trichloramine formation is also dependent on concentrations of the cyanurate species that hypochlorite targets for nucleophilic attack. The electrostatic potentials (ESP) of key cyanurate species modeled by DFT reveal that increasing chlorine substitution renders the triazine ring more electron deficient, and therefore more susceptible to attack by a nucleophile (Table S8). However, above pH 8.5 at these Cl:Cy ratios, the dominant chlorocyanurate species is monochlorinated HClCy^–^ (Table S5). Particularly at pH 9.2 with the Cl:Cy ratios employed here, Cl_2_Cy^–^ comprises <5% of total chlorine, and other di- or trichlorinated species comprise less than 0.01%. Equation 2 reflects the likely contribution of HClCy^–^ in this reaction pathway:

2 $${\mathrm{HClCy}}^{-}+8{\mathrm{ClO}}^{-}+4H_{2}O\to 3{\mathrm{NCl}}_{3}+2{\mathrm{CO}}_{2}+{{\mathrm{HCO}}_{3}}^{-}+8{\mathrm{OH}}^{-}$$

At pH 9.2 with chlorine held constant, dichlor is expected to have ∼50% higher total chlorocyanurate concentrations than trichlor (Table S5). This may explain why dichlore formed the most trichloramine, despite trichlore being expected to have a ∼13% higher hypochlorite concentration.

The trend in maximum trichloramine concentrations at pH 9.2 (dichlor > trichlor > monochlor) is generally consistent with the trend in HAN concentrations observed in the DBP formation experiments with the fall and summer samples at pH ∼8.2–8.3 (triclor ≅ dichlor > monochlor > chlorine). However, chlorocyanurates also promoted HAN formation in the lower pH DBP formation experiments. At pH 7.3, hypochlorite concentrations comprise only ∼15%, 7.0%, and 1.4% of the total chlorine in trichlor, dichlor and monochlor formulations, respectively. However, concentrations of Cl_2_Cy^–^ are notably higher at low pH, comprising as much as 25% and 30% of the total chlorine in trichlor and dichlor, respectively. Thus, the hypochlorite-mediated trichloramine formation pathway may still occur at low pH, at concentrations too low to detect by our method.

Additionally, another potential low-pH pathway for cyanurate decomposition to trichloramine is oxidative cleavage by hydroxyl radicals, previously demonstrated in the context of advanced oxidation processes.^70^ Chlorination of NOM can form hydroxyl radicals,^71^ which might be sufficient to decompose some cyanurate to trichloramine, contributing to HAN formation. To test the principle that hydroxide radicals can increase HAN formation in real waters as a function of cyanuric acid concentration, the fall water was supplemented with low ammonia (0.2 mg/L as N) to induce breakpoint chlorination, which rapidly occurs at Cl:N molar ratios above ∼1.5 and generates hydroxyl radicals.^72^ Trichloramine is formed during breakpoint chlorination in the absence of cyanuric acid,^40^ but if hydroxyl radicals cleave the triazine ring to form trichloramine, additional HAN formation should be observed in chlorocyanurate conditions compared to chlorine-only.

Breakpoint chlorination promoted greater increases in HAN concentrations in all three chlorocyanurate conditions compared to chlorine (Figure S4). Dichlor produced the greatest increase in HAN concentrations, which was >150% greater than the increase in HAN concentrations with chlorine-only. This was followed by monochlor, which exhibited >100% greater increase in HANs than observed with chlorine-only, even though monochlor had the lowest effective Cl:N molar ratio (2.2) and thus presumably lower hydroxyl radical exposure. A controlled experiment confirmed that breakpoint chlorination with dichlor formed higher initial trichloramine concentrations than with chlorine and exhibited much slower decay (Figure 4B).

One interpretation of these results is that some excess trichloramine was produced by triazine ring cleavage by hydroxyl radicals, as proposed above. However, these results could also be explained by the difference in free chlorine concentrations; although the same amount of total chlorine was added, the free chlorine-to-nitrogen ratio for dichlor was ∼3:1 compared to 14:1 for chlorine. Higher Cl:N ratios speed breakpoint reactions, and free chlorine release by chlorocyanurate may be a rate limiting step within the reaction cascade. Ultimately, the potential for excessive trichloramine concentrations with chlorocyanurate disinfection is a concern for DBP formation and toxicity and warrants further investigation.

### Implications

This study is the first to prove that chlorocyanurates are active participants in DBP formation reactions. The findings underscore the value of measuring multiple DBP classes in order to evaluate alternative disinfectants. Compared to conventional chlorine, chlorocyanurates produced lower levels of regulated DBPs (e.g., THM4, chlorinated HAAs), but significantly promoted brominated and nitrogenated DBPs, resulting in 1.5–2x higher calculated toxicity. Several distinctive features of chlorocyanurate chemistry were demonstrated that point to risk trade-offs and operational considerations for long-term drinking water disinfection. First, caution should be exercised in substituting chlorocyanurate disinfection for conventional chlorine in waters with elevated bromide levels. Avoiding moderate bromide (e.g., ∼100 μg/L) can be difficult, particularly in low-resource settings. However, full-scale utilities should consider this in their decision-making. Use of higher Cl:Cy molar ratios (i.e., trichlor) could minimize brominated DBP formation. Future research is needed to confirm the role of bromocyanurates and identify more specific engineering controls for minimizing brominated DBPs in chlorocyanurate systems.

Second, chlorocyanurate disinfection promoted greater HAN formation and higher trichloramine concentrations than conventional chlorine under all conditions tested. High Cl:Cy ratios in high pH waters greatly enhanced trichloramine formation and therefore HAN formation, due to higher concentrations of hypochlorite and monochlorinated cyanurate species. On the other hand, low Cl:Cy ratios may produce too little free chlorine to protect against pathogens, along with promoting brominated DBPs. Given the effect of Cl:Cy ratio, we hypothesized that the order of addition of chlorine and cyanuric acid may be key to avoid excessive HAN formation at high Cl:Cy ratios, similar to strategies for avoiding breakpoint chlorination in ammoniacal waters.^73^ We tested this by comparing two methods of dichlor preparation: 1) spiking concentrated chlorine into dilute cyanuric acid (the method used in this study) or 2) mixing 50/50 dilute chlorine and dilute cyanuric acid. Little difference was observed; maximum trichloramine concentrations were 277 μM with concentrated chlorine and 271 μM with dilute reagents, with slightly faster trichloramine formation in the latter (Figure S5). Mixing dilute reagents in equal volume may better approximate commercial dichlor tablet products, in which the Cl:Cy ratio at the tablet interface is exactly 2:1. However, other differences may be incurred by tablet dissolution dynamics; this is beyond the scope of the present manuscript. A study comparing liquid and solid chlorocyanurate dosing is warranted. That said, the minimal difference between mixing conditions is notable, and reminiscent of the relatively consistent findings regarding THM formation with chlorocyanurates across studies using commercial tablet products^74,75^ and liquid reagents mixed at the bench.^45^

Future research should evaluate the effect of chlorocyanurate disinfection on more DBP classes. For example, I-DBPs could be promoted by cyanuric acid addition if iodide engages in nucleophilic substitution with chlorocyanurates in the same manner as bromide. Regardless, concern over DBPs should not overshadow the potential benefits of chlorocyanurate disinfection, particularly in point-of-use and short-term emergency scenarios where shelf life and ease of use are key and chronic DBP exposure risk is low relative to pathogen risk in untreated water. There may also be benefits associated with chlorocyanurates in full-scale applications. For example, previous authors suggested that chlorocyanurates may offer a more stable chlorine residual than conventional chlorine. Although no significant difference in the residuals was observed in this study, each experiment targeted the same total chlorine residual. Chlorine decay rates are a function of chorine concentration,^76^ such that differences between chlorine and chlorocyanurates might emerge at higher or lower doses, or as a function of another variable such as temperature. Alternative disinfectants that provide the disinfection efficacy of chlorine with slower residual decay would be advantageous to guard against pathogen re-entry in distribution systems, or to decelerate chlorine decay during extreme heat events.^15^ Research is underway to evaluate whether chlorocyanurates offer these benefits.

## Supplementary Material





## Notes

Additional detail on materials and methods; additional tables and figures providing results of supporting experiments and detailed modeling results (PDF)
Experimental data generated (XLSX)

## References

[1] Drinking Water, 2024. CDC. https://www.cdc.gov/drinking-water/about/how-water-treatment-works.html (accessed 2025–02–14).

[2] Hoff J. C., Akin E. W. (1986). Microbial
Resistance to Disinfectants: Mechanisms and
Significance. Environ. Health Perspect..

[3] Richardson S. D., Plewa M. J., Wagner E. D., Schoeny R., Demarini D. M. (2007). Occurrence,
Genotoxicity, and Carcinogenicity of Regulated and Emerging Disinfection
by-Products in Drinking Water: A Review and Roadmap for Research. Mutat. Res..

[4] Richardson S. D., Kimura S. Y. (2020). Water Analysis:
Emerging Contaminants and Current Issues. Anal.
Chem..

[5] Villanueva C. M., Cantor K. P., Grimalt J. O., Malats N., Silverman D., Tardon A., Garcia-Closas R., Serra C., Carrato A., Castaño-Vinyals G., Marcos R., Rothman N., Real F. X., Dosemeci M., Kogevinas M. (2006). Bladder Cancer
and Exposure to Water Disinfection By-Products through Ingestion,
Bathing, Showering, and Swimming in Pools. Am.
J. Epidemiol..

[6] Villanueva C. M., Cordier S., Font-Ribera L., Salas L. A., Levallois P. (2015). Overview of
Disinfection By-Products and Associated Health Effects. Curr. Environ. Health Rep..

[7] Furst K. E., Coyte R. M., Wood M., Vengosh A., Mitch W. A. (2019). Disinfection
Byproducts in Rajasthan, India: Are Trihalomethanes a Sufficient Indicator
of Disinfection Byproduct Exposure in Low-Income Countries?. Environ. Sci. Technol..

[8] Furst K. E. (2025). Quantitative
Evaluation of Regulatory Indicators for Brominated Haloacetic Acids
in Drinking Water. Environ. Sci. Technol..

[9] Wagner E. D., Plewa M. J. (2017). CHO Cell Cytotoxicity
and Genotoxicity Analyses of
Disinfection By-Products: An Updated Review. J. Environ. Sci. China.

[10] Mao Y., Zhang L., Dong H. (2018). Formation of Trihalomethanes in Swimming
Pool Waters Using Sodium Dichloroisocyanurate as an Alternative Disinfectant. Water Sci. Technol..

[11] Mazhar M. A., Khan N. A., Ahmed S., Khan A. H., Hussain A., Rahisuddin, Changani F., Yousefi M., Ahmadi S., Vambol V. (2020). Chlorination Disinfection
By-Products in Municipal Drinking Water – A Review. J. Clean. Prod..

[12] Hua G., Reckhow D. A. (2007). Comparison of Disinfection Byproduct Formation from
Chlorine and Alternative Disinfectants. Water
Res..

[13] Furst K. E., Pecson B. M., Webber B. D., Mitch W. A. (2018). Tradeoffs
between
Pathogen Inactivation and Disinfection Byproduct Formation during
Sequential Chlorine and Chloramine Disinfection for Wastewater Reuse. Water Res..

[14] Furst K. E., Bolorinos J., Mitch W. A. (2021). Use of Trihalomethanes
as a Surrogate
for Haloacetonitrile Exposure Introduces Misclassification Bias. Water Res. X.

[15] Furst K. E., Graham K. E., Weisman R. J., Adusei K. B. (2024). It’s
Getting
Hot in Here: Effects of Heat on Temperature, Disinfection, and Opportunistic
Pathogens in Drinking Water Distribution Systems. Water Res..

[16] Zhang W., DiGiano F. A. (2002). Comparison of Bacterial Regrowth in Distribution Systems
Using Free Chlorine and Chloramine: A Statistical Study of Causative
Factors. Water Res..

[17] Curling E. A., McKie M. J., Meteer L., Saunders B., Andrews S. A., Andrews R. C. (2022). Estimation of Chloramine
Decay in Drinking Water Distribution
Systems. J. Water Process Eng..

[18] Wahman D. G. (2018). Chlorinated
Cyanurates: Review of Water Chemistry and Associated Drinking Water
Implications. J. Am. Water Works Assoc..

[19] Clancey V.
J. (1975). Fire Hazards
of Calcium Hypochlorite. J. Hazard. Mater..

[20] Lantagne D. S., Blount B. C., Cardinali F., Quick R. (2008). Disinfection By-Product
Formation and Mitigation Strategies in Point-of-Use Chlorination of
Turbid and Non-Turbid Waters in Western Kenya. J. Water Health.

[21] Smith D. W., Sultana S., Crider Y. S., Islam S. A., Swarthout J. M., Goddard F. G. B., Rabbani A., Luby S. P., Pickering A. J., Davis J. (2021). Effective Demand for In-Line Chlorination Bundled with Rental Housing
in Dhaka, Bangladesh. Environ. Sci. Technol..

[22] Plan review policy for dichlor and trichlor installations (DDW-Eng-0032 April 27, 2017). Utah Division of Drinking Water. https://lf-public.deq.utah.gov/WebLink/DocView.aspx?id=437765&eqdocs=DDW-2024-009554 (accessed 2025–05–08).

[23] Canelli E. (1974). Chemical,
Bacteriological, and Toxicological Properties of Cyanuric Acid and
Chlorinated Isocyanurates as Applied to Swimming Pool Disinfection:
A Review. Am. J. Public Health.

[24] Khazaei A., Sarmasti N., Yousefi
Seyf J., Merati Z. (2020). Anchoring N-Halo (Sodium
Dichloroisocyanurate) on the Nano-Fe3O4 Surface as “Chlorine
Reservoir”: Antibacterial Properties and Wastewater Treatment. Arab. J. Chem..

[25] Wahman D. G., Alexander M. T., Dugan A. G. (2019). Chlorinated Cyanurates in Drinking
Water: Measurement Bias, Stability, and Disinfectant Byproduct Formation. AWWA Water Sci..

[26] Clasen T., Edmondson P. (2006). Sodium Dichloroisocyanurate
(NaDCC) Tablets as an Alternative
to Sodium Hypochlorite for the Routine Treatment of Drinking Water
at the Household Level. Int. J. Hyg. Environ.
Health.

[27] Wahman D. G. (2018). First Acid
Ionization Constant of the Drinking Water Relevant Chemical Cyanuric
Acid from 5 to 35 °c. Environ. Sci. Water
Res. Technol..

[28] Fitzgerald G. P., DerVartanian M. E. (1969). Pseudomonas Aeruginosa for the Evaluation of Swimming
Pool Chlorination and Algicides. Appl. Microbiol..

[29] Yamashita T., Sakae K., Ishihara Y., Isomura S., Inoue H. (1988). Virucidal
Effect of Chlorinated Water Containing Cyanuric Acid. Epidemiol. Infect..

[30] Feldstein, C. M. ; Rickabaugh, J. ; Miltner, R. Effect of Cyanuric Acid, a Chlorine Stabilizer, on Trihalomethane Formation; EPA/600/D-84/167 (NTIS PB84209105); U.S. Environmental Protection Agency, 1984. https://cfpub.epa.gov/si/si_public_record_report.cfm?Lab=NRMRL&dirEntryId=36623.

[31] Dong S., Masalha N., Plewa M. J., Nguyen T. H. (2017). Toxicity of Wastewater
with Elevated Bromide and Iodide after Chlorination, Chloramination,
or Ozonation Disinfection. Environ. Sci. Technol..

[32] Regli S., Chen J., Messner M., Elovitz M. S., Letkiewicz F. J., Pegram R. A., Pepping T. J., Richardson S. D., Wright J. M. (2015). Estimating Potential Increased Bladder Cancer Risk
Due to Increased Bromide Concentrations in Sources of Disinfected
Drinking Waters. Environ. Sci. Technol..

[33] Yang Y., Komaki Y., Kimura S. Y., Hu H.-Y., Wagner E. D., Mariñas B. J., Plewa M. J. (2014). Toxic Impact of Bromide and Iodide
on Drinking Water Disinfected with Chlorine or Chloramines. Environ. Sci. Technol..

[34] Yang L., Schmalz C., Zhou J., Zwiener C., Chang V. W.-C., Ge L., Wan M. P. (2016). An Insight
of Disinfection By-Product
(DBP) Formation by Alternative Disinfectants for Swimming Pool Disinfection
under Tropical Conditions. Water Res..

[35] Lantagne D. S., Cardinali F., Blount B. C. (2010). Disinfection By-Product Formation
and Mitigation Strategies in Point-of-Use Chlorination with Sodium
Dichloroisocyanurate in Tanzania. Am. J. Trop.
Med. Hyg..

[36] Salas L. A., Cantor K. P., Tardon A., Serra C., Carrato A., Garcia-Closas R., Rothman N., Malats N., Silverman D., Kogevinas M., Villanueva C. M. (2013). Biological
and Statistical Approaches
for Modeling Exposure to Specific Trihalomethanes and Bladder Cancer
Risk. Am. J. Epidemiol..

[37] Jeong C. H., Postigo C., Richardson S. D., Simmons J. E., Kimura S. Y., Mariñas B. J., Barcelo D., Liang P., Wagner E. D., Plewa M. J. (2015). Occurrence
and Comparative Toxicity of Haloacetaldehyde
Disinfection Byproducts in Drinking Water. Environ.
Sci. Technol..

[38] Falk, R. A. The Chlorine/Cyanuric Acid Relationship and Implications for Nitrogen Trichloride, 2019. https://f.hubspotusercontent10.net/hubfs/5079918/Scientific%20Documents/Trichloramine-CYA-Relationships%2C%20Richard%20Falk.pdf (accessed 2025–02–14).

[39] Wojtowicz, J. A. Oxidation of Cyanuric Acid with Hypochlorite. Journal of the Swimming Pool and Spa Industry, 2011, 4, 23–29. https://www.poolhelp.com/wp-content/uploads/2017/05/JSPSI_V4N2_pp23-28.pdf (accessed 2024–02–05).

[40] Huang H., Zheng H., Jiao J., Lei Y., Zhou Y., Qiu J., Yang X. (2022). Trichloramine and Hydroxyl Radical Contributions to
Dichloroacetonitrile Formation Following Breakpoint Chlorination. Environ. Sci. Technol..

[41] Chuang Y.-H., Szczuka A., Mitch W. A. (2019). Comparison
of Toxicity-Weighted Disinfection
Byproduct Concentrations in Potable Reuse Waters and Conventional
Drinking Waters as a New Approach to Assessing the Quality of Advanced
Treatment Train Waters. Environ. Sci. Technol..

[42] Khorasani H., Xu J., Nguyen T., Kralles Z., Westerhoff P., Dai N., Zhu Z. (2021). Contribution of Wastewater- versus Non-Wastewater-Derived
Sources to Haloacetonitriles Formation Potential in a Wastewater-Impacted
River. Sci. Total Environ..

[43] Xu J., Kralles Z. T., Hart C. H., Dai N. (2020). Effects of Sunlight
on the Formation Potential of Dichloroacetonitrile and Bromochloroacetonitrile
from Wastewater Effluents. Environ. Sci. Technol..

[44] Chuang Y. H., Shi H. J. (2022). UV/Chlorinated Cyanurates as an Emerging Advanced Oxidation
Process for Drinking Water and Potable Reuse Treatments. Water Res..

[45] Wahman D. G., Alexander M. T., Dugan A. G. (2019). Chlorinated Cyanurates
in Drinking
Water: Measurement Bias, Stability, and Disinfectant Byproduct Formation. AWWA Water Sci..

[46] Krupińska I. (2020). Aluminium
Drinking Water Treatment Residuals and Their Toxic Impact on Human
Health. Mol. Basel Switz..

[47] Furst K. E., Smith D. W., Bhatta L. R., Islam M., Sultana S., Rahman M., Davis J., Mitch W. A. (2022). Effects of Intrusion
on Disinfection Byproduct Formation in Intermittent Distribution Systems. ACS EST Water.

[48] Standard methods for the examination of water and wastewater, 2023. American Public Health Association, American Water Works Association, and Water Environment Federation. https://www.standardmethods.org/pb-assets/downloads/SM%20SOP-2023-2.0-1676654450900.pdf (accessed 2024–12–01).

[49] Falk R. A., Blatchley E. R., Kuechler T. C., Meyer E. M., Pickens S. R., Suppes L. M. (2019). Assessing the Impact of Cyanuric Acid on Bather’s
Risk of Gastrointestinal Illness at Swimming Pools. Water.

[50] Wahman D.
G., Alexander M. T. (2019). A Drinking
Water Relevant Water Chemistry Model for
the Free Chlorine and Cyanuric Acid System from 5 to 35 °C. Environ. Eng. Sci..

[51] Zeng T., Plewa M. J., Mitch W. A. (2016). N-Nitrosamines
and Halogenated Disinfection
Byproducts in U.S. Full Advanced Treatment Trains for Potable Reuse. Water Res..

[52] Furst K. E., Pecson B. M., Webber B. D., Mitch W. A. (2018). Distributed
Chlorine
Injection To Minimize NDMA Formation during Chloramination of Wastewater. Environ. Sci. Technol. Lett..

[53] Li X.-F., Mitch W. A. (2018). Drinking Water Disinfection
Byproducts (DBPs) and Human
Health Effects: Multidisciplinary Challenges and Opportunities. Environ. Sci. Technol..

[54] Lau S. S., Wei X., Bokenkamp K., Wagner E. D., Plewa M. J., Mitch W. A. (2020). Assessing
Additivity of Cytotoxicity Associated with Disinfection Byproducts
in Potable Reuse and Conventional Drinking Waters. Environ. Sci. Technol..

[55] Lu T. (2024). A Comprehensive
Electron Wavefunction Analysis Toolbox for Chemists, Multiwfn. J. Chem. Phys..

[56] Lu T., Chen F. (2012). Multiwfn: A Multifunctional
Wavefunction Analyzer. J. Comput. Chem..

[57] Miller M.
P., McKnight D. M. (2010). Comparison
of Seasonal Changes in Fluorescent Dissolved
Organic Matter among Aquatic Lake and Stream Sites in the Green Lakes
Valley. J. Geophys. Res. Biogeosciences.

[58] Plewa M. J., Muellner M. G., Richardson S. D., Fasano F., Buettner K. M., Woo Y.-T., McKague A. B., Wagner E. D. (2008). Occurrence, Synthesis,
and Mammalian Cell Cytotoxicity and Genotoxicity of Haloacetamides:
An Emerging Class of Nitrogenous Drinking Water Disinfection Byproducts. Environ. Sci. Technol..

[59] Hua G., Reckhow D. A. (2012). Evaluation of Bromine
Substitution Factors of DBPs
during Chlorination and Chloramination. Water
Res..

[60] Premarathna S. M., Gunasekera V., Sathasivan A. (2023). Modelling
the Effect of Bromide on
Chlorine Decay in Raw and Coagulated Surface Waters. J. Water Process Eng..

[61] Bloomfield S. F., Miles G. A. (1979). The Antibacterial Properties of Sodium
Dichloroisocyanurate
and Sodium Hypochlorite Formulations. J. Appl.
Bacteriol..

[62] Kumar K., Shinness R. W., Margerum D. W. (1987). Kinetics
and mechanisms of the base
decomposition of nitrogen trichloride in aqueous solution. Inorg. Chem..

[63] Sanderson, W. D. Sealed Biocide Composition and Sealed Biocide Article. U.S. Patent US2008/0299161 A1, 2008. https://patentimages.storage.googleapis.com/d2/91/ac/4ad563c250f8de/AU2006333074A1.pdf (accessed 2025–13–05).

[64] Paterson, L. O. Halogenated Cyanuric Acids and Their Salts. U.S. Patent US3147254A, 1964. https://patentimages.storage.googleapis.com/04/91/ea/c156749a1462fd/US3147254.pdf (accessed 2025–09–09).

[65] de
Almeida L. S., Esteves P. M., de Mattos M. C. (2006). A New Regioselective
Bromination of Activated Aromatic Rings. Synthesis.

[66] Ersan G., Ersan M. S., Kanan A., Karanfil T. (2021). Predictive Modeling
of Haloacetonitriles under Uniform Formation Conditions. Water Res..

[67] Mezyk S. P., Rogalski M. H., Dang A. N., Bartels D. M., Hardison D. R., Cooper W. J. (2025). Radical Treatment of Haloacetonitriles
in Aqueous Systems:
A Kinetic Study. ACS EST Water.

[68] Carlson, R. H. Sodium Hypochlorite Treatment for Removal of Cyanurate Compounds from Aqueous Waste Streams. U.S Patent US4075094A, February 21, 1978. https://patents.google.com/patent/US4075094/en (accessed 2025–05–16).

[69] Chuang Y.-H., Chen T.-Y., Chou C.-S., Chu L.-K., Hou C.-Y., Szczuka A. (2023). Critical Role of Trichloramine Interaction with Dichloramine
for N-Nitrosamine Formation during Breakpoint Chlorination. Environ. Sci. Technol..

[70] Chuang Y.-H., Shi H.-J. (2022). UV/Chlorinated Cyanurates
as an Emerging Advanced Oxidation
Process for Drinking Water and Potable Reuse Treatments. Water Res..

[71] Rodríguez E. M., von Gunten U. (2020). Generation of Hydroxyl Radical during
Chlorination
of Hydroxyphenols and Natural Organic Matter Extracts. Water Res..

[72] Chuang Y.-H., Chou C.-S., Chu Y.-L. (2024). Unveiling the Critical
Pathways of
Hydroxyl Radical Formation in Breakpoint Chlorination: The Role of
Trichloramine and Dichloramine Interactions. Environ. Sci. Technol..

[73] Furst K. E., Pecson B. M., Webber B. D., Mitch W. A. (2018). Distributed Chlorine
Injection to Minimize NDMA Formation during Chloramination of Wastewater. Environ. Sci. Technol. Lett..

[74] Lantagne D. S., Cardinali F., Blount B. C. (2010). Disinfection By-Product Formation
and Mitigation Strategies in Point-of-Use Chlorination with Sodium
Dichloroisocyanurate in Tanzania. Am. J. Trop.
Med. Hyg..

[75] Lantagne D. S., Blount B. C., Cardinali F., Quick R. (2008). Disinfection By-Product
Formation and Mitigation Strategies in Point-of-Use Chlorination of
Turbid and Non-Turbid Waters in Western Kenya. J. Water Health.

[76] Powell J. C., Hallam N. B., West J. R., Forster C. F., Simms J. (2000). Factors Which
Control Bulk Chlorine Decay Rates. Water Res..

