# Supplementary material for: Enhanced Formation of Brominated and Nitrogenous Disinfection Byproducts in Drinking Water Disinfection with Chlorocyanurates
Source: Environ Sci Technol. 2025 Dec 19;60(1):1357–67. doi: 10.1021/acs.est.5c07394 (PMC12810233; doi:10.1021/acs.est.5c07394)
Supplement: Supplementary file 1 [file es5c07394_si_001.pdf]

Supporting Information:

Enhanced formation of brominated and nitrogenous disinfection byproducts in drinking water disinfection with chlorocyanurates

Kadmiel B. Adusei[1,2], Hafiz Usama Tanveer[1], Zachary T. Kralles[3], Kirin Emlet Furst[1,2*]

[1] Charles E. Via, Jr. Department of Civil and Environmental Engineering, Occoquan Watershed Monitoring Laboratory, Virginia Polytechnic Institute and State University, Manassas, Virginia, 20110

[2] Sid and Reva Dewberry Department of Civil, Environmental and Infrastructure Engineering, George Mason University, Fairfax, Virginia, 22030

[3] Department of Environmental Health and Engineering, Johns Hopkins University, Baltimore, Maryland, 21205

* Corresponding author: Charles E. Via, Jr. Department of Civil and Environmental Engineering, Occoquan Watershed Monitoring Laboratory, Virginia Polytechnic Institute and State University, Manassas, Virginia, 20110; (703)361-5606; kfurst@vt.edu

Summary: 22 pages, 4 texts, 11 tables, 5 figures.

TABLE OF CONTENTS

Text S1: Reagents and reference standards 4

Text S2: Instrumental analysis...4

Text S3: Methods for spectroscopic analysis and trichloramine quantitation 5

Text S4: Cyanuric acid decay in chlorocyanurate solution at high pH 6

Tables..7

Table S1. Water quality characteristics of surface water samples prior to amendment with bromide..7

Table S2. Reference standards and reagents, with vendor information and manufacturer reported purity. 8

Table S3. Instrument retention time (RT) and Multiple Reaction Monitoring (MRM) transitions, including those used for quantitation (t1) and confirmation (t2) for all DBPs analyzed. Volatiles and haloacetic acids (HAAs) were analyzed using different methods. 9

Table S4. Method detection limits (MDLs), method reporting limits (MRLs), and mean matrix spike recoveries for low (10 ng/L) and high (100 ng/L) analyte spike concentrations in DI water. 10

Table S5. Distribution of chlorinated species as percent total chlorine for each experiment at 22℃, calculated using the equilibrium model as reported by Wahman et al. (2018a)................................11

Table S6: Source water quality parameters, experimental conditions, chlorine residuals, and disinfection byproduct (DBP) concentrations measured in all surface water SDS experiments.........12

Table S7: Synthetic water quality parameters, experimental conditions, chlorine residuals, and disinfection byproduct (DBP) concentrations measured in all synthetic water SDS experiments......12

Table S8. Electrostatic potential (ESP) and polarity-related descriptors for four chlorocyanurate species. Values were computed from the molecular ESP surface using Multiwfn. Electrostatic potential surfaces were generated using density functional theory (DFT) with the def2-SVP basis set in ORCA. 13

Table S9: Molar extinction coefficients ($M^{-1}cm^{-1}$) used in this study for free and combined chlorine species, extracted from Chuang et al. (2023) and confirmed experimentally for OCl^- and HOCl......14

Table S10: Maximum trichloramine (NCl_3) concentrations (µM) and conservatively derived molar yield estimates from total cyanuric acid (Cy) and total chlorine (Cl_2) with the indicated reagent concentrations at pH 9.2; details of the calculations are provided Text S3.......................................15

Table S11: Summary of average free, total, and combined chlorine concentrations (mg/L as Cl_2) in surface water samples under SDS conditions. 16

Figures 17

Figure S1. The equilibria that forms among free chlorine, cyanuric acid and its chlorinated derivatives; modified from Brady et al., (1963) and Wahman, (2018). 17

Figure S2. Volatile DBPs formed in summer 2023 samples following treatment with 0.21 mM chlorine only, or in the presence of increasing cyanuric acid concentrations targeting 3:1, 2:1 and 1:1 chlorine

to cyanuric acid molar ratios, on a concentration basis with A) low (0.04 mg/L) bromide or B) 100 μg/L additional bromide, and toxicity-weighted DBP concentrations with C) low or D) high bromide. 18

Figure S3. DBPs formed in the winter 2023 samples with 0.96 mg/L bromide, following treatment with 0.21 mM chlorine only, or in the presence of increasing cyanuric acid concentrations targeting 3:1, 2:1 and 1:1 chlorine to cyanuric acid molar ratios, as A) weight-based concentrations or B) toxicity-weighted concentrations. 19

Figure S4. Difference in HAN concentrations for each disinfectant condition between the fall sample with no ammonia and the fall sample amended with ammonia (0.2 mg/L as N), followed by disinfection with 0.21 mM chlorine-only or with cyanuric acid in Cl:Cy molar ratios of 3:1 (trichlor), 2:1 (dichlor), and 1:1 (monochlor). Effective Cl:N molar ratios based on predicted free available chlorine are 15 (chlorine-only), 8.4 (trichlor), 5.7 (dichlor), and 2.2 (monochlor) as calculated with the equilibrium model. 20

Figure S5: Comparison of trichloramine concentrations (μM) in two different reagent mixing scenarios for dichlor (prepared in DI water, pH 9.2, 18 mM NaOCl), in which concentrated NaOCl was spiked into dilute cyanuric acid (“concentrated”), or dilute NaOCl and cyanuric acid were prepared individually in 50/50 volume ratio and dilute cyanuric acid was slowly poured into dilute NaOCl (“dilute”). 21

References 22

Text S1: Reagents and reference standards

Analytical standards for priority disinfection byproducts (DBPs) were purchased or custom synthesized at the highest purity available from Sigma-Aldrich (St. Louis, MO, USA), CanSyn Chem. Corp. (Toronto, Ontario, Canada), and Accustandard Inc. (New Haven, CT, USA). All volatile and haloacetic acid (HAA) standard mixes were prepared in methyl tert-butyl ether (MTBE). The M-551.1B mix, which includes dichloroacetonitrile (DCAN), bromochloroacetonitrile (BCAN), dibromoacetonitrile (DBAN), trichloroacetonitrile (TCAN), trichloronitromethane (TCNM), 1,1,1-trichloropropanone (1,1,1-TCP), trichloroacetaldehyde (TCAL), and 1,1-dichloropropanone (1,1-DCP), was purchased from Accustandard. The trihalomethanes mix (M-501) was also purchased from Accustandard and prepared in MTBE. Dibromochloroacetaldehyde (DBCAL) and bromodichloroacetaldehyde (BDCAL) were purchased from CanSyn. Trichloroacetaldehyde (TCAL) was obtained from Accustandard, and tribromoacetaldehyde (TBAL) was purchased from Sigma-Aldrich. Nine HAA standards, i.e. bromodichloroacetic acid (BDCAA), chloroacetic acid (CAA), bromoacetic acid (BAA), dibromoacetic acid (DBAA), dichloroacetic acid (DCAA), bromochloroacetic acid (BCAA), chlorodibromoacetic acid (CDBAA), trichloroacetic acid (TCAA), and tribromoacetic acid (TBAA), were obtained from Accustandard (USA). Vendor information, percentage purity, and abbreviations for each standard are provided in Table S2.

Text S2: Instrumental analysis

The study utilized a gas chromatography triple quadrupole (GCTQ) Mass Spectrometer (MS) system, consisting of an Agilent 8890 GC and 7010B MS with a high-efficiency electron ionization source (Agilent Technologies, Santa Clara, CA). The Rtx-200MS GC column (30 m × 0.25 mm ID × 0.25 µm film thickness) from Restek Corporation (Bellefonte, PA, USA) was used. One microliter of sample was injected with the inlet in splitless mode. The temperature program for HAAs commenced with an initial temperature of 35°C, which was maintained for 0.1 minutes. The temperature was then elevated to 220°C at a rate of 360°C per minute and held for 5 minutes. Subsequently, the temperature was increased to 280°C at a rate of 720°C per minute. For volatiles and semi-volatiles, the GC oven temperature was initiated at 35°C and maintained for 5 minutes. It was then increased to 200°C at a rate of 9°C per minute and ramped to 280°C, where it was held for 20 minutes. The MS transfer line temperature was maintained at 225°C. The source temperature and the quadrupole temperature were held constant at 200°C and 150°C, respectively. The MS was operated with an ionization energy of 70 eV. Optimal Multiple Reaction Monitoring (MRM) transitions were determined using the Optimizer Program as part of the Agilent Mass Hunter software. Peaks were confirmed by matching spectra of known standards to the NIST database where available, or spectra were added to an internal database based on peaks of known standards. Two MRM transitions were selected for each compound, one for quantitation (t1) and one for confirmation (t2). Retention times and MRM parameters for each compound are reported in Table S3.

Text S3: Methods for spectroscopic analysis and trichloramine quantitation

Trichloramine analysis by UV-Vis spectroscopy with absorbance at 336 nm and/or 360 nm is the most widely reported method in the literature (e.g., Chuang et al., 2023; Schurter et al., 1995). Trichloramine molar absorptivity at 336 nm (195 $M^{-1}cm^{-1}$) is higher than at 360 nm (130 $M^{-1}cm^{-1}$), but OCl^- also has notable absorbance at 336 nm and at 360 nm given the higher concentrations of OCl- compared to trichloramine.

To evaluate absorbance at 336 nm and 360 nm by other species in solution, several controls were performed. First, separate solutions of sodium hypochlorite (NaOCl) and cyanuric acid in buffered, deionized (DI) water were analyzed at 336 nm and 360 nm (as well as other wavelengths). We confirmed that OCl^- absorbs at 336 and 360 nm, respectively, in NaOCl solution of pH 9.2, with molar absorptivities reported of ε_{336}= 71.4 $M^{-1}cm^{-1}$ and ε_{360} = 10.6 $M^{-1}cm^{-1}$ (Chuang et al., 2023). Cyanuric acid/cyanurate absorbance was zero at both wavelengths, as expected from prior literature (O'Brien et al., 1974). Regarding other possible species that might form in our experiments, we ruled out other inorganic chloramines, dichloramine and monochloramine, which have negligible molar absorptivity above 330 nm (Gendel and Lahav, 2012). Finally, we ruled out interference from chlorocyanurates by noting that in monochlor solutions with low Cl:Cy molar ratios (1:1) and at low pH, negligible absorbance at these wavelengths was present, despite the known presence of multiple chlorocyanurate species. It is therefore reasonable to conclude that chlorocyanurates do not absorb at these wavelengths.

Thus, OCl- is the only species of concern for interference with trichloramine absorption at 336 or 360 nm. We accounted for this in trichloramine concentration by using the system of equations for NCl3 and OCl- absorbance at 336 and 360 nm (*Eq S1, S2*) and molar exctinction coefficients provided in Table S9:

$$\text{Eq. S1.} \qquad abs_{336} = \varepsilon_{336,NCl3}[NCl_3] + \varepsilon_{336,OCl}[OCl^-]$$

$$\text{Eq. S2.} \qquad abs_{360} = \varepsilon_{360,NCl3}[NCl_3] + \varepsilon_{360,OCl}[OCl^-]$$

For quality assurance, to test the accuracy of this method and ensure that trichloramine concentrations are not overestimated, several calculations were done with a chlorine control. First, the concentrations of OCl- and trichloramine were calculated for an NaOCl solution of 18 mM, in which trichloramine should be zero. The OCl- concentration calculated following the above method was highly accurate, at 18.1 mM, and the nominal trichloramine concentration was slightly positive at 0.016 mM. Thus, we rounded up to establish a practical method reporting limit (MRL) of 0.020 mM for trichloramine.

The molar yields of trichloramine from cyanuric acid and chlorine were estimated from the maximum concentrations of trichloramine formed in each experiment. The reagent concentrations, maximum trichloramine concentrations, and molar yield of trichloramine calculated for each experiment is provided in Table S10. These yields are likely an underestimate, as the maximum trichloramine concentration does not account for auto-decay of trichloramine in solution nor loss through volatilization (though the latter was minimized by immediately capping the cuvette).

Ultimately, the goal of this analysis was to provide an initial estimate of the potential for trichloramine formation in chlorocyanurate solutions, and to evaluate the effect of Cl:Cy ratio.

Text S4: Cyanuric acid decay in chlorocyanurate solution at high pH

To confirm that trichloramine formation coincides with decomposition of chlorocyanurates, the decay of total cyanuric acid was measured using the melamine-induced turbidity method (Hach Model CY-3). A trichlorocyanurate (3:1) solution containing an initial concentration of 86 mg/L cyanuric acid was stored in the dark at room temperature for ~24 hours, to coincide with the experimental methods used for DBP formation. The final cyanuric acid concentration was ~35 mg/L, a loss of 59%. This result may be exaggerated by the high concentrations required for accurate determination by the melamine-induced turbidity method.

Tables

Table S1. Water quality characteristics of surface water samples prior to amendment with bromide.

**Water source**	**Condition**	**pH**	**Cond. (µS/cm)**	**DOC (mg/L)**	**UV_{254}**	**$SUVA_{254}$ (L/mg-m)**	**Source bromide (mg/L)**
Surface water	Summer 2023	8.3	0.27	5.5	0.27	4.9	0.04
Surface water	Winter 2023	7.3	1.1	5.0	0.23	4.6	0.96
Surface Water	Fall 2024	8.4	0.42	8.0	0.10	1.3	0.05

Table Key: Cond.: Conductivity; SUVA: specific ultraviolet absorbance; DOC: dissolved organic carbon.

Table S2. Reference standards and reagents, with vendor information and manufacturer reported purity.

Compound/Reagents	Abbr.	Vendor	Purity (%)
**(THM) Trihalomethanes**			
Trichloromethane	TCM	Accustandard	99.2%
Bromochloromethane	BDCM	Accustandard	100.0
Dibromochloromethane	DBCM	Accustandard	98.4%
Tribromomethane	TBM	Accustandard	99.2%
**HALs (Haloacetaldehydes)**			
Trichloroacetaldehyde	TCAL	Accustandard	95%
Bromodichloroacetaldehyde	BDCAL	Accustandard	93%
Dibromochloroacetaldehyde	DBCAL	Accustandard	87%
Tribromoacetaldehyde	TBAL	Accustandard	97%
**HANs (Haloacetonitrile)**			
Trichloroacetonitrile	TCAN	Accustandard	98.0%
Dichloroacetonitrile	DCAN	Accustandard	99.4%
Bromochloroacetonitrile	BCAN	Accustandard	97.5%
Dibromoacetonitrile	DBAN	Accustandard	99.1%
**HKs (Haloketones)**			
1,1,1-Trichloro-2-propanone	1,1,1-TCP	Accustandard	98.7%
1,1-Dichloro-2-propanone	1,1-DCP	Accustandard	95.5%
**HNMs (Halonitromethanes)**			
Trichloronitromethane	TCNM	Accustandard	98.0%
**HAAs (Haloacetic Acids)**			
Bromoacetic acid	BAA	Accustandard	100.0
Bromochloroacetic acid	BCAA	Accustandard	99.0%
Chloroacetic acid	CAA	Accustandard	99.0%
Dibromochloroacetic acid	DBAA	Accustandard	90.0%
Dichloroacetic acid	DCAA	Accustandard	100.0
Trichloroacetic acid	TCAA	Accustandard	99.3%
Dibromochloroacetic acid	DBCAA	Accustandard	97.0%
Bromodichloroacetic acid	BDCAA	Accustandard	97.8%
Dalapon	Dalapon	Accustandard	96.7%
Tribromoacetic acid	TBAA	Accustandard	99.2%
**Internal standard**			
1, 2 Dibromopropane	1,2BDP	Accustandard	98%
**Surrogate**			
2 Bromobutanoic acid	2-BBA	Accustandard	97%
**Solvent**			
Methyl tert-butyl ether	MTBE	Fisher scientific	99%
Methanol	MeOH	Sigma Aldrich	99.9%

Table S3. Instrument retention time (RT) and Multiple Reaction Monitoring (MRM) transitions, including those used for quantitation (t1) and confirmation (t2) for all DBPs analyzed. Volatiles and haloacetic acids (HAAs) were analyzed using different methods.

**DBP**	**RT (min)**	**MRM transition (t_1) quant (m/z)**	**MRM transition (t_2) qual (m/z)**
**Volatiles**			
TBM	6.72	171>92	254>173
TCM	2.68	83>48	85>49
DBCM	4.92	127>48	208>129
BDCM	3.40	83>48	85>47
DBAN	9.18	118>91	120>93
DCAN	5.23	74>47	74>39
BCAN	7.28	74>47	153>74
TCAN	3.75	108>73	110>73
TCNM	6.00	117>82	119>84
TCAL	4.59	111>83	82>47
1,1,1-TCP	8.17	97>61	125>43
1,1-DCP	5.92	91>63	83>48
TBAL	10.0	174>93	171.7>92.
BDCAL	6.50	111>83	83>47
DBCAL	8.38	129>48	128>47
**HAAs**			
BAA	4.93	121>93	123>95
BCAA	6.40	127>48	129>48
CAA	3.90	77>49	108>76
DBAA	8.20	173>92	175>94
DCAA	4.89	83>47	83>48
TCAA	5.80	117>82	119>84
DBCAA	9.46	209>128	207>128
BDCAA	7.90	161>82	163>82
TBAA	10.44	253>172	251>172

Table S4. Method detection limits (MDLs), method reporting limits (MRLs), and mean matrix spike recoveries for low (10 ng/L) and high (100 ng/L) analyte spike concentrations in DI water.

**Analyte**	**MDL (µg/L)**	**MRL (µg/L)**	**% recovery (10 ng/L)**	**% recovery (100 ng/L)**
**THMs**				
TCM	0.001	0.003	133%	99%
BDCM	0.001	0.003	123%	99%
DBCM	0.001	0.003	126%	100%
TBM	0.001	0.003	118%	99%
**HALs**				
TCAL	0.001	0.003	118%	94%
BDCAL	0.005	0.014	81%	123%
DBCAL	0.002	0.007	74%	103%
TBAL	0.003	0.008	70%	108 %
**HANs**				
TCAN	0.001	0.003	114%	92%
DCAN	0.001	0.003	125%	98%
BCAN	0.001	0.003	78%	110%
DBAN	0.006	0.017	72%	88%
**HNMs**				
TCNM	0.001	0.003	104%	98%
**HKs**				
1,1,1-TCP	0.003	0.009	116%	89%
1,1-DCP	0.001	0.004	129%	95%
**HAAs**				
BAA	0.002	0.005	75%	71%
BCAA	0.002	0.005	85%	71%
CAA	0.001	0.002	88%	75%
DBAA	0.002	0.006	82%	75%
DCAA	0.002	0.005	91%	75%
TCAA	0.002	0.005	88%	75%
DBCAA	0.002	0.005	80%	82%
BDCAA	0.002	0.006	80%	84%
TBAA	0.002	0.007	80%	75%

Table S5. Distribution of chlorinated species as percent total chlorine for each experiment at 22℃, calculated using the equilibrium model as reported by Wahman et al. (2018a).

**Sample**	**Disinfectant**	**Cl_2:Cy (mol/mol)**	**pH**	**[HOCl]**	**[OCl-]**	**[Cl_3Cy]**	**[Cl_2Cy^-]**	**[HCl_2Cy]**	**[$ClCy^{2-}$]**	**[$HClCy^-$]**	**[H_2ClCy]**
Summer 2023	Chlorine	---	8.3	15.5%	84.6%	---	---	---	---	---	---
	Trichlor	3.0	8.3	8.20%	44.7%	0.00%	15.2%	0.00%	0.13%	16.8%	0.02%
	Dichlor	2.0	8.3	5.22%	28.5%	0.00%	17.7%	0.00%	0.24%	30.8%	0.03%
	Monochlor	1.0	8.3	1.73%	9.42%	0.00%	12.2%	0.00%	0.51%	64.0%	0.06%
Winter 2023	Chlorine	---	7.3	64.8%	35.3%	---	---	---	---	---	---
	Trichlor	3.0	7.3	27.3%	14.9%	0.00%	24.8%	0.00%	0.01%	8.22%	0.08%
	Dichlor	2.0	7.3	12.7%	6.92%	0.00%	29.6%	0.00%	0.02%	21.1%	0.20%
	Monochlor	1.0	7.3	2.60%	1.42%	0.00%	17.4%	0.00%	0.05%	60.7%	0.57%
Fall 2024	Chlorine	---	8.4	12.7%	87.4%	---	---	---	---	---	---
	Trichlor	3.0	8.4	6.95%	47.7%	0.00%	13.7%	0.00%	0.18%	17.9%	0.01%
	Dichlor	2.0	8.4	4.57%	31.4%	0.00%	16.0%	0.00%	0.32%	31.8%	0.02%
	Monochlor	1.0	8.4	1.63%	11.2%	0.00%	11.5%	0.00%	0.63%	63.7%	0.05%
Synthetic water (pH 7.3)	Chlorine	---	7.3	64.8%	35.3%	---	---	---	---	---	---
	Trichlor	3.0	7.3	27.3%	14.9%	0.00%	24.8%	0.00%	0.01%	8.22%	0.08%
	Dichlor	2.0	7.3	12.7%	6.92%	0.00%	29.6%	0.00%	0.02%	21.1%	0.20%
	Monochlor	1.0	7.3	2.60%	1.42%	0.00%	17.4%	0.00%	0.05%	60.7%	0.57%
Synthetic water (pH 9.2)	Chlorine	---	9.2	2.26%	97.9%	---	---	---	---	---	---
	Trichlor	3.0	9.2	1.60%	69.3%	0.00%	3.64%	0.00%	1.30%	20.6%	0.00%
	Dichlor	2.0	9.2	1.30%	56.4%	0.00%	4.51%	0.00%	1.97%	31.4%	0.00%
	Monochlor	1.0	9.2	0.80%	34.5%	0.00%	4.60%	0.00%	3.29%	52.4%	0.01%

Table S6: Source water quality parameters, experimental conditions, chlorine residuals, and disinfection byproduct (DBP) concentrations measured in all surface water SDS experiments.

[See “Supplemental Tables S6 and S7.xlsx.”]

Table S7: Synthetic water quality parameters, experimental conditions, chlorine residuals, and disinfection byproduct (DBP) concentrations measured in all synthetic water SDS experiments.

[See “Supplemental Tables S6 and S7.xlsx.”]

Table S8. Electrostatic potential (ESP) and polarity-related descriptors for four chlorocyanurate species. Values were computed from the molecular ESP surface using Multiwfn. Electrostatic potential surfaces were generated using density functional theory (DFT) with the def2-SVP basis set in ORCA.

**Descriptors**	**Cl_3Cy**	**Cl_2Cy^-**	**$HClCy^-$**	**$ClCy^{2-}$**
Average ESP (kcal/mol)	10,139.90	9,059.90	7,901.80	7,901.30
Min ESP (kcal/mol)	8,415.50	7,630.70	6,357.00	6,290.50
Max ESP (kcal/mol)	12,746.40	11,232.00	9,777.60	9,683.80
Internal charge separation (Π)	909	760.6	656.8	618.7
Molecular Polarity index (MPI, eV)	439.7	392.9	342.7	342.6
Polar Surface Area (Å²)	191.3	171.3	155.7	151.2

Table S9: Molar extinction coefficients ($M^{-1}cm^{-1}$) used in this study for free and combined chlorine species, extracted from Chuang et al. (2023) and confirmed experimentally for OCl^- and HOCl.

**Wavelength**	**HOCl**	**OCl^-**	**NH_2Cl**	**$NHCl_2$**	**NCl_3**
**294**	101	365	27.0	272	160
**336**	3.0	71.4	0.0	35.0	195
**360**	2.3	10.6	0.0	3.9	130

Table S10: Maximum trichloramine (NCl_3) concentrations (μM) and conservatively derived molar yield estimates from total cyanuric acid (Cy) and total chlorine (Cl_2) with the indicated reagent concentrations at pH 9.2; details of the calculations are provided Text S3.

**Solution composition**	**Cyanuric acid (mM)**	**Chlorine (mM)**	Max NCl_3 (μM)	**Molar yield NCl_3/Cy**	**Molar yield NCl_3/Cl_2**
**Cyanuric acid**	20	N/A	0.0	0.0%	0.0%
**Chlorine**	N/A	20	0.0	0.0%	0.0%
**Trichlor**	6.7	20	230	3.4%	1.2%
**Dichlor**	13	20	277	2.1%	1.4%
**Monochlor**	20	20	109	0.55%	0.55%

Table S11: Summary of average free, total, and combined chlorine concentrations (mg/L as Cl_2) in surface water samples under SDS conditions.

**Bromide (mg/L)**	**Disinfectant**	**Free chlorine**	**Total chlorine**	**Combined chlorine**
Summer samples				
0.04	Free Cl	1.17	1.24	0.07
	Monochlor	1.28	1.34	0.06
	Dichlor	1.07	1.15	0.08
	Trichlor	1.07	1.12	0.05
0.14	Free Cl	1.19	1.26	0.07
	Monochlor	1.28	1.36	0.08
	Dichlor	1.10	1.2	0.10
	Trichlor	1.10	1.13	0.03
Winter samples				
0.96	Free Cl	1.24	1.34	0.10
	Monochlor	1.38	1.45	0.07
	Dichlor	1.34	1.40	0.06
	Trichlor	1.21	1.24	0.03
Fall samples				
0.05	Free Cl	0.61	0.79	0.18
	Monochlor	0.60	0.63	0.03
	Dichlor	0.58	0.64	0.06
	Trichlor	0.51	0.58	0.07
0.15	Free Cl	0.52	0.60	0.07
	Monochlor	0.80	0.95	0.15
	Dichlor	0.59	0.71	0.12
	Trichlor	0.53	0.58	0.05

Figures

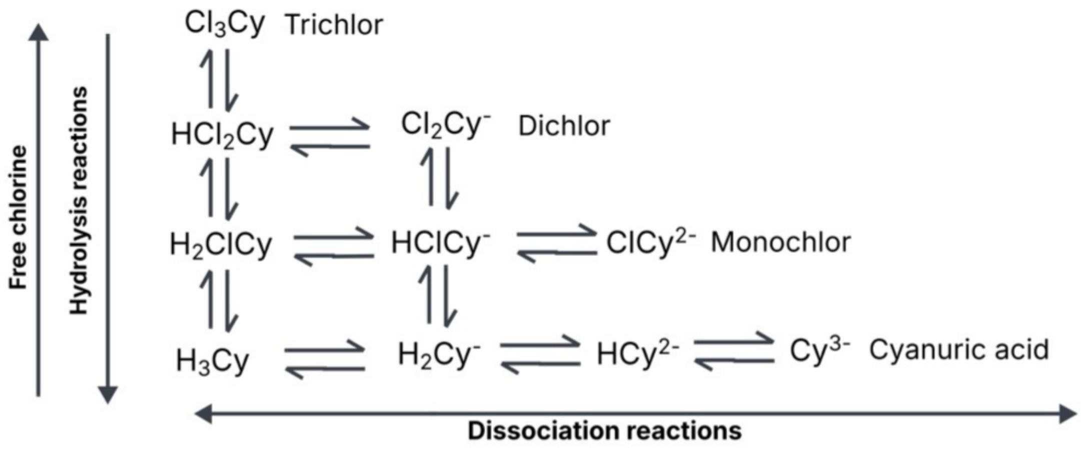

Figure S1. The equilibria that forms among free chlorine, cyanuric acid and its chlorinated derivatives; modified from Brady et al., (1963) and Wahman, (2018).

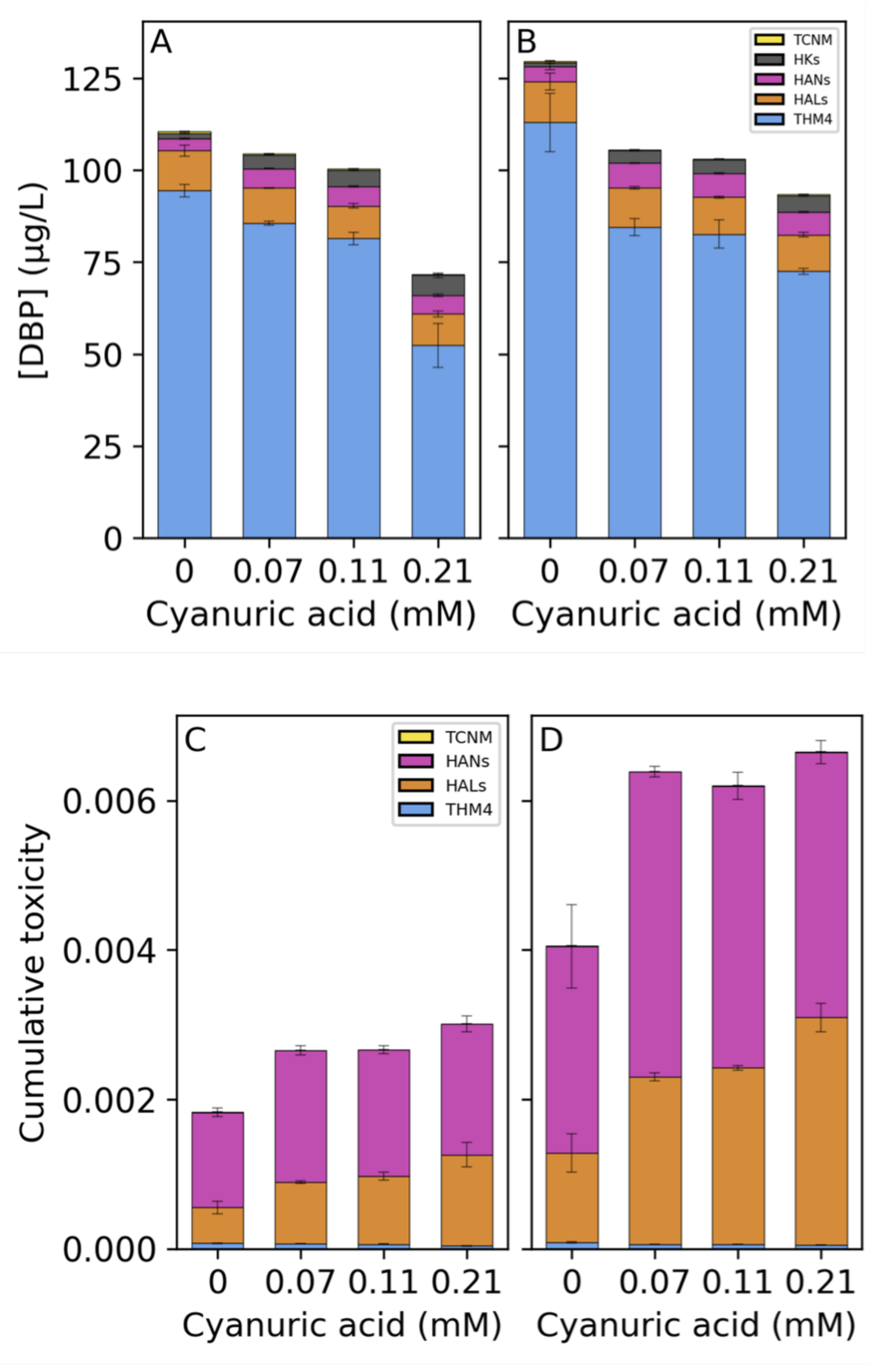

Figure S2. Volatile DBPs formed in summer 2023 samples following treatment with 0.21 mM chlorine only, or in the presence of increasing cyanuric acid concentrations targeting 3:1, 2:1 and 1:1 chlorine to cyanuric acid molar ratios, on a concentration basis with A) low (0.04 mg/L) bromide or B) 100 µg/L additional bromide, and toxicity-weighted DBP concentrations with C) low or D) high bromide.

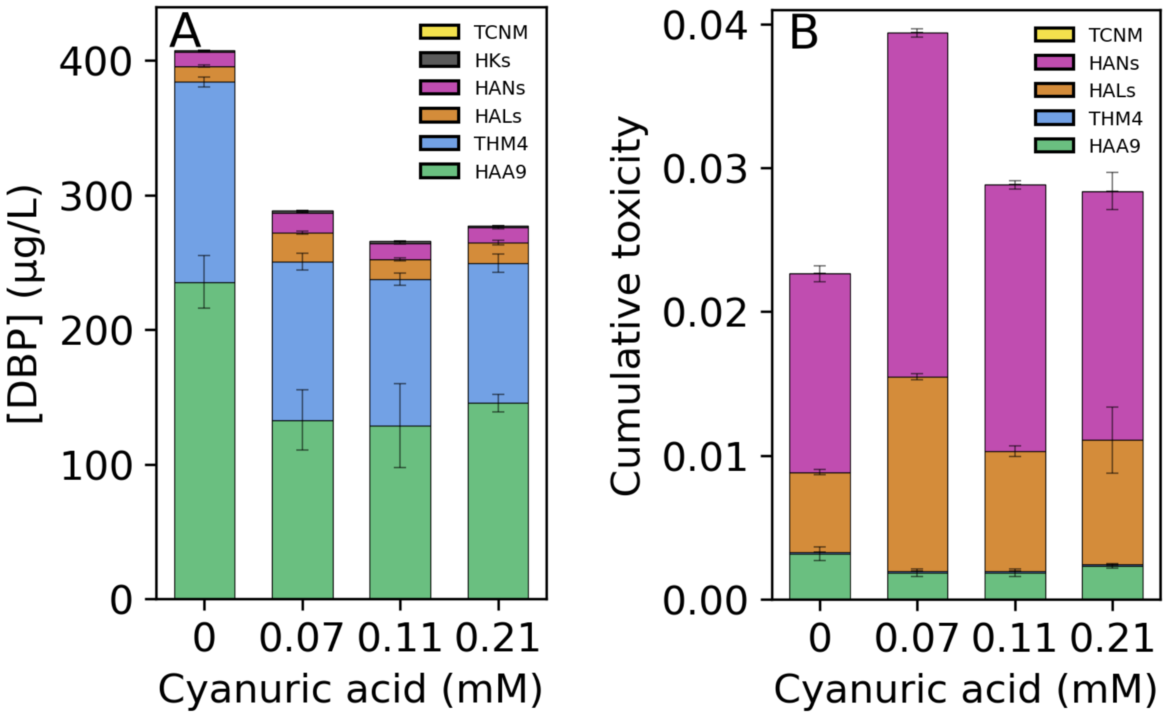

Figure S3. DBPs formed in the winter 2023 samples with 0.96 mg/L bromide, following treatment with 0.21 mM chlorine only, or in the presence of increasing cyanuric acid concentrations targeting 3:1, 2:1 and 1:1 chlorine to cyanuric acid molar ratios, as A) weight-based concentrations or B) toxicity-weighted concentrations.

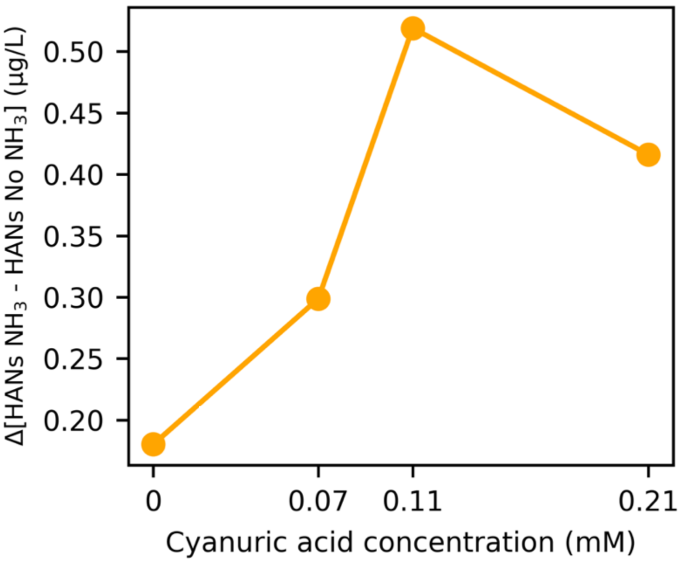

Figure S4. Difference in HAN concentrations for each disinfectant condition between the fall sample with no ammonia and the fall sample amended with ammonia (0.2 mg/L as N), followed by disinfection with 0.21 mM chlorine-only or with cyanuric acid in Cl:Cy molar ratios of 3:1 (trichlor), 2:1 (dichlor), and 1:1 (monochlor). Effective Cl:N molar ratios based on predicted free available chlorine are 15 (chlorine-only), 8.4 (trichlor), 5.7 (dichlor), and 2.2 (monochlor) as calculated with the equilibrium model.

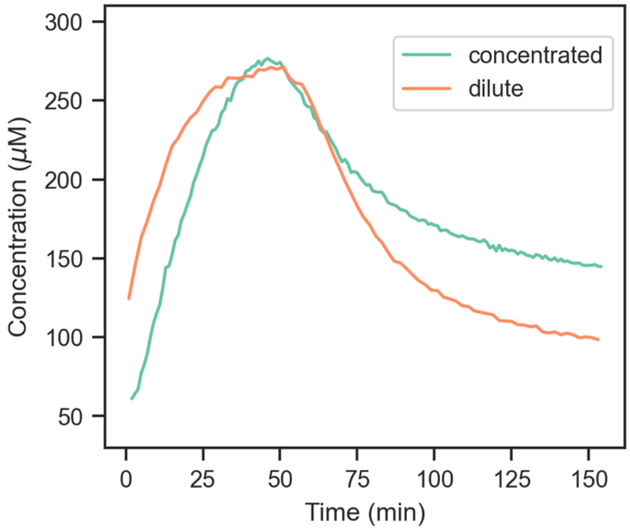

Figure S5: Comparison of trichloramine concentrations (μM) in two different reagent mixing scenarios for dichlor (prepared in DI water, pH 9.2, 18 mM NaOCl), in which concentrated NaOCl was spiked into dilute cyanuric acid (“concentrated”), or dilute NaOCl and cyanuric acid were prepared individually in 50/50 volume ratio and dilute cyanuric acid was slowly poured into dilute NaOCl (“dilute”).

References

Brady, A.P., Sancier, K.M., Sirine, Gloria., 1963. Equilibria in Solutions of Cyanuric Acid and its Chlorinated Derivatives. J. Am. Chem. Soc. 85, 3101–3104. https://doi.org/10.1021/ja00903a011

Chuang, Y.-H., Chen, T.-Y., Chou, C.-S., Chu, L.-K., Hou, C.-Y., Szczuka, A., 2023. Critical Role of Trichloramine Interaction with Dichloramine for *N* -Nitrosamine Formation during Breakpoint Chlorination. Environ. Sci. Technol. 57, 15232–15242. https://doi.org/10.1021/acs.est.3c03326

Gendel, Y., Lahav, O., 2012. Revealing the mechanism of indirect ammonia electrooxidation. Electrochimica Acta 63, 209–219. https://doi.org/10.1016/j.electacta.2011.12.092

O'Brien, J.E., J. C., M., Butler, J.N., 1974. Equilibria in Aqueous Solutions of Chlorinated Isocyanurate [WWW Document]. URL https://www.troublefreepool.com/~richardfalk/pool/OBrien.htm (accessed 9.1.25).

Schurter, L.M., Bachelor, P.P., Margerum, D.W., 1995. Nonmetal Redox Kinetics: Mono-, Di-, and Trichloramine Reactions with Cyanide Ion. Environ. Sci. Technol. 29, 1127–1134. https://doi.org/10.1021/es00004a035

Wahman, D.G., 2018. Chlorinated Cyanurates: Review of Water Chemistry and Associated Drinking Water Implications. J. AWWA 110. https://doi.org/10.1002/awwa.1086
